# Regulation of Zn and Fe transporters by the *GPC1* gene during early wheat monocarpic senescence

**DOI:** 10.1186/s12870-014-0368-2

**Published:** 2014-12-19

**Authors:** Stephen Pearce, Facundo Tabbita, Dario Cantu, Vince Buffalo, Raz Avni, Hans Vazquez-Gross, Rongrong Zhao, Christopher J Conley, Assaf Distelfeld, Jorge Dubcovksy

**Affiliations:** Department of Plant Sciences, University of California, Davis, CA 95616 USA; Consejo Nacional de Investigaciones Científicas y Técnicas and Instituto de Recursos Biológicos, CIRN, INTA, N. Repetto y Los Reseros s/n (1686), Hurlingham, Argentina; Department of Viticulture and Enology, University of California, Davis, CA 95616 USA; Faculty of Life Sciences, Department of Molecular Biology and Ecology of Plants, Tel Aviv University, Tel Aviv, 69978 Israel; Department of Plant Nutrition, College of Resources and Environmental Science, China Agricultural University, Beijing, 100193 People’s Republic of China; Department of Statistics, University of California, Davis, CA 95616 USA; Faculty of Life Sciences, Department of Molecular Biology and Ecology of Plants, Tel Aviv University, Tel Aviv, 69978 Israel; Howard Hughes Medical Institute and Gordon & Betty Moore Foundation Investigator, Davis, CA 95616 USA

**Keywords:** Wheat, Senescence, GPC, Zinc transport, Iron transport, ZIP

## Abstract

**Background:**

During wheat senescence, leaf components are degraded in a coordinated manner, releasing amino acids and micronutrients which are subsequently transported to the developing grain. We have previously shown that the simultaneous downregulation of *Grain Protein Content* (*GPC*) transcription factors, *GPC1* and *GPC2*, greatly delays senescence and disrupts nutrient remobilization, and therefore provide a valuable entry point to identify genes involved in micronutrient transport to the wheat grain.

**Results:**

We generated loss-of-function mutations for *GPC1* and *GPC2* in tetraploid wheat and showed in field trials that *gpc1* mutants exhibit significant delays in senescence and reductions in grain Zn and Fe content, but that mutations in *GPC2* had no significant effect on these traits. An RNA-seq study of these mutants at different time points showed a larger proportion of senescence-regulated genes among the *GPC1* (64%) than among the *GPC2* (37%) regulated genes. Combined, the two *GPC* genes regulate a subset (21.2%) of the senescence-regulated genes, 76.1% of which are upregulated at 12 days after anthesis, before the appearance of any visible signs of senescence. Taken together, these results demonstrate that *GPC1* is a key regulator of nutrient remobilization which acts predominantly during the early stages of senescence. Genes upregulated at this stage include transporters from the *ZIP* and *YSL* gene families, which facilitate Zn and Fe export from the cytoplasm to the phloem, and genes involved in the biosynthesis of chelators that facilitate the phloem-based transport of these nutrients to the grains.

**Conclusions:**

This study provides an overview of the transport mechanisms activated in the wheat flag leaf during monocarpic senescence. It also identifies promising targets to improve nutrient remobilization to the wheat grain, which can help mitigate Zn and Fe deficiencies that afflict many regions of the developing world.

**Electronic supplementary material:**

The online version of this article (doi:10.1186/s12870-014-0368-2) contains supplementary material, which is available to authorized users.

## Background

In annual grasses, monocarpic senescence is the final stage of a plant’s development during which vegetative tissues are degraded and their cellular nutrients and amino acids are transported to the developing grain. The regulation of this process is crucial for the plant’s reproductive success and determines to a large extent the nutritional quality of the harvested grain. Among wild diploid relatives of wheat, there exists large variation in Zn and Fe grain content, whereas modern wheat germplasm collections exhibit comparatively lower and less variable Zn and Fe concentrations [1,2], demonstrating that improvements in these traits are possible. Zn and Fe deficiency afflict many parts of the developing world where wheat constitutes a major part of the diet, making the development of nutritionally-enhanced wheat varieties an important target for breeders tackling this problem [3].

The main source of protein and micronutrients in the wheat grain is the flag leaf and, to a lesser extent, the lower leaves [4,5]. When applied to the leaf tip, radioactively-labelled Zn is efficiently translocated to the developing wheat grain [6]. The close correlation between Zn and Fe content in the grain suggests some level of redundancy in the regulatory mechanisms used by the plant to transport these micronutrients [1]. However, the regulation of gene expression associated with nutrient transport from leaves to grain during wheat monocarpic senescence is poorly understood. A detailed understanding of these mechanisms will be required in order to engineer wheat varieties with improved nutritional quality through biofortification [7].

Several studies in other species, including barley, rice and Arabidopsis have revealed distinct mechanisms regulating micronutrient transport in vegetative tissues, which are described below according to their sub-cellular location.

### Transport between chloroplast and cytoplasm

Because of its importance to photosynthesis, Fe is particularly abundant within the chloroplasts, which harbor ~90% of all Fe in the leaf during vegetative development [8]. Therefore, the remobilization of Fe from the chloroplast is an important process during monocarpic senescence. In Arabidopsis a member of the ferric chelate reductase (*FRO*) gene family is highly expressed in photosynthetic tissues and localizes to the chloroplast membrane, suggestive of a role in the reduction-based import of Fe into the chloroplasts [9]. In rice, certain *FRO* genes are preferentially expressed in the leaf vasculature rather than the roots, suggesting that this may be a conserved transport mechanism [10]. Certain members of the Heavy Metal ATPase (HMA) family of transporters have been implicated in the reverse process; nutrient export from the chloroplast to the cytoplasm. In Arabidopsis, *AtHMA1* localizes to the chloroplast membrane and facilitates Zn export from the chloroplast [11] and in barley, *HvHMA1* facilitates both Zn and Fe export from the chloroplast [12].

### Transport between vacuole and cytoplasm

Additional mechanisms within the leaf exist to facilitate Fe and Zn transport between the vacuole and cytoplasm as part of a sequestration strategy, since high concentrations of either nutrient can be toxic for the plant cell. In rice, two *VACUOLAR IRON TRANSPORTER* genes, *OsVIT1* and *OsVIT2*, encode proteins which are localized to the vacuolar membrane (tonoplast) and facilitate Zn^2+^ and Fe^2+^ import to the vacuole [13]. Likewise, the *ZINC-INDUCED FACILITATOR-LIKE* (ZIFL) genes encode Zn-transporters which are implicated in vacuole transport. In Arabidopsis, *ZIF1* localizes to the tonoplast and *zif1* mutants accumulate Zn in the cytosol, suggesting that these transporters promote vacuolar sequestration of Zn by facilitating its import into the vacuole [14]. However, several of the thirteen *ZIFL* genes recently described in rice are induced in the flag leaves during senescence [15]. This suggests that in monocots, certain *ZIFL* genes may also play a role in promoting nutrient remobilization during senescence. The NRAMP family of transporters appears to regulate nutrient export from the vacuole. In Arabidopsis, *NRAMP3* and *NRAMP4* are induced in Fe-deficient conditions and plants combining mutations in both these genes fail to mobilize vacuolar reserves of Fe [16].

### Transport from cytoplasm to phloem

For their transport to the grain, micronutrients must be transported from the cytoplasm across the plasma membrane to be loaded into the phloem. This process is facilitated by members of the Yellow stripe like (YSL) and ZRT, IRT like protein (ZIP) families of membrane-bound transporters, which transport metal-chelate complexes across the plasma membrane in the leaves of several plant species [17-19]. In Arabidopsis, two Fe-transporting members of the *YSL* gene family were shown to be essential for normal seed development [20] and in barley, *HvZIP7* knockout mutant plants exhibit significantly reduced Zn levels in the grain, suggesting that this family may also be important for nutrient loading into the phloem [21].

**Figure 1:**
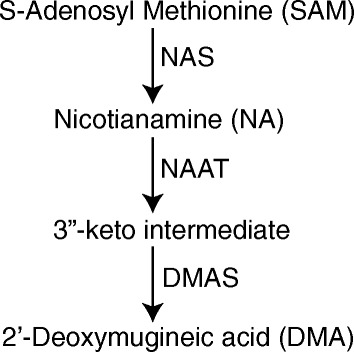
**Biosynthesis of mugienic acid phytosiderophores.** The combination of three molecules of SAM to form one molecule of NA is catalyzed by NAS. NA is converted to DMA through the action of NAAT to form a 3”-keto intermediate and then by DMAS to form DMA. Adapted from Bashir et al. [26].

Because Zn and Fe ions exhibit limited solubility in the alkaline environment of the phloem, they are transported in association with a chelator [19]. Nicotianamine (NA) is one such important chelator and is a member of the mugineic acid family phytosiderophores [22]. NA biosynthesis is regulated by the enzyme nicotianamine synthase (NAS) by combining three molecules of S-Adenosyl Methionine [23], and can be further catalyzed to 2’-deoxymugineic acid (DMA) by the sequential activity of nicotianamine aminotransferase (NAAT) [24,25], which generates a 3”-keto intermediate and DMA synthase (DMAS, Figure 1) [26]. Although Zn has been shown to associate with DMA in the rice phloem [27], a recent study suggests that it is more commonly associated with NA [28]. In contrast, the principal chelator of Fe in the rice phloem is DMA [29]. It has been hypothesized that phloem transport represents the major limiting factor determining Zn and Fe content of cereal grains [30] and this is supported by several studies which demonstrate that altering *NAS* expression can have significant impacts on Zn and Fe grain and seed content. In Arabidopsis, plants carrying non-functional mutations in all *NAS* genes exhibit low Fe levels in sink tissues, while maintaining high levels in ageing leaves [31]. Conversely, *NAS* overexpression results in the accumulation of higher concentrations of Zn and Fe in Arabidopsis seed [32], rice grains [33,34] and barley grains [35].

#### Regulation of senescence and nutrient translocation

Monocarpic senescence and nutrient translocation to the grain occur simultaneously, requiring a precise coordination of these two processes. This is reflected in the large-scale transcriptional changes in the plant’s vegetative tissues during the onset of senescence, as documented in recent expression studies in Arabidopsis [36,37], barley [38] and wheat [39,40]. These studies consistently identify increased expression levels of a number of transcription factors of different classes. Particularly important roles have been identified for members of the NAC family [38,41-44]. In wheat, one such NAC-domain transcription factor, *Grain Protein Content 1* (*GPC1*, also known as *NAM1*), has been shown to play a critical role in the regulation of both the rate of senescence and the levels of protein, Zn and Fe in the mature grain [44].

Originally identified as a QTL which enhances grain protein content in wild emmer (*Triticum turgidum* spp. *dicoccoides*) [45], the genomic region of chromosome arm 6BS including *GPC1* was later shown to also accelerate senescence in tetraploid and hexaploid wheat [44,46,47]. A paralogous gene, *GPC2* (also known as *NAM2*), was identified on chromosome arm 2BS, which shares 91% similarity with *GPC1* at the DNA level [44]. Transcripts of *GPC1* and *GPC2* are first detected in flag leaves shortly before anthesis and increase rapidly during the early stages of senescence. In hexaploid wheat, plants transformed with a *GPC*-RNAi construct targeting all homologous *GPC* genes and plants carrying loss-of-function mutations in all *GPC1* homoeologs, both exhibit a three-week delay in the onset of senescence as well as significant reductions in the transport of amino acids (N), Zn and Fe to the grain [5,44,46]. Therefore, *GPC* mutants represent an excellent tool to dissect the mechanisms underlying Zn and Fe transport from leaves to grains during monocarpic senescence.

In the current study, we used RNA-seq to identify genes differentially regulated in the flag leaves during three early stages of monocarpic senescence in tetraploid wheat. We also identified genes that were differentially expressed within each of these stages between tetraploid WT and *gpc* mutants, which exhibited reduced Zn and Fe grain concentrations. We identified members of different transporter families, which were differentially regulated both during the early stages of senescence and between genotypes with different *GPC* alleles. Results from this study define more precisely the role of individual *GPC* genes in the regulation of transporter gene families in senescing leaves and identify new differentially regulated targets for Fe and Zn biofortification strategies in wheat.

## Results

### *GPC1* and *GPC2* mutations and their effect on senescence and nutrient translocation

**Figure 2:**
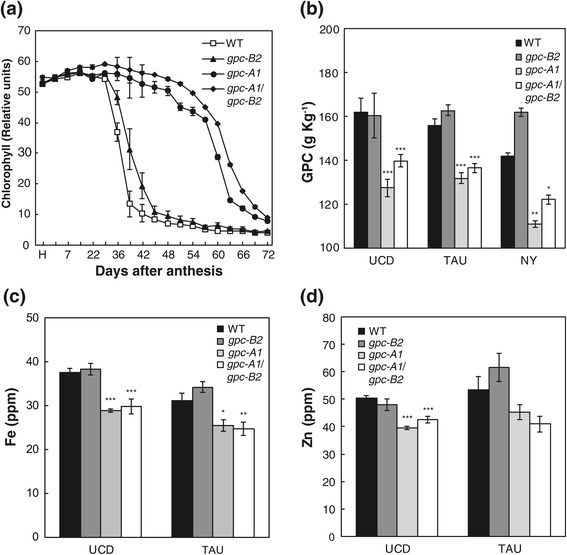
***GPC*** **mutations in tetraploid wheat result in significant delays in senescence and reductions in protein, Zn and Fe content in the grain. (a)** Relative chlorophyll content of flag leaves taken from the UCD-2012 field experiment **(b)** GPC content of mature grains harvested from three experiments, (UCD n = 10, TAU and NY n = 4) **(c)** Fe and **(d)** Zn content of mature grains harvested from UCD-2012 and TAU-2012 experiments (n = 5). * = P < 0.5, ** = P < 0.01, *** = P < 0.001, difference when compared to WT control sample from Dunnett’s test. UCD = UC Davis 2012 experiment, TAU = Tel Aviv University 2012 experiment, NY = Newe Ya’ar research center 2012 experiment.

Field experiments comparing wild type (WT), single (*gpc-A1* and *gpc-B2*), and double (*gpc-A1/gpc-B2*) mutants showed consistent results across the four tested environments (UCD-2012, TAU-2012, NY-2012 and NY-2013, Figure 2, Additional file 1: Figure S1 and S2). None of the *gpc* mutants showed significant differences in heading time relative to the WT, which is consistent with the known upregulation of the *GPC* genes after anthesis [44]. Both the *gpc-A1* and *gpc-A1/gpc-B2* mutants were associated with a significant delay in senescence relative to the WT and the *gpc-B2* mutant. In the Davis field experiment (UCD-2012), these two mutants showed a 27-day delay in the onset of senescence in comparison to WT plants (Figure 2a), and consistent results were observed in field experiments carried out in Tel Aviv and Newe Ya’ar (Additional file 1: Figure S1). The differences in senescence observed between WT and *gpc-B2* or between *gpc-A1* and *gpc-A1/gpc-B2* mutants were comparatively much smaller (Figure 2a).

To test the effects of the *GPC* mutations on yield components in a tetraploid background, we measured thousand kernel weight (TKW) in three field environments and dry spike weight in the Davis field experiment. We detected a marginally significant reduction in TKW associated with the *gpc-A1* and *gpc-A1/gpc-B2* mutant genotypes (*P* =0.02, Additional file 1: Figure S2a). These mutant genotypes were also associated with significant reductions in dry spike weight in the Davis field experiment which was lower in both *gpc-A1* and *gpc-A1/gpc-B2* mutants at 35 DAA (*P* <0.001) and in the *gpc-A1/gpc-B2* mutant at 42 and 49 DAA (*P* <0.001, Additional file 1: Figure S2b).

The delays in the onset of senescence in the *gpc-A1* and *gpc-A1/gpc-B2* mutants relative to WT plants were associated with reductions in protein, Zn and Fe levels in the mature grain (Figure 2, b-d). Similarly, the marginal differences in senescence between WT and *gpc-B2* or between *gpc-A1* and *gpc-A1/gpc-B2* mutants (Figure 2a) were paralleled by the absence of significant differences in protein, Zn and Fe levels in the grain in the different field experiments (Figure 2, b-d). Similar reductions in GPC were observed across the different field experiments (Figure 2b), which ranged between 19.5% (WT *vs. gpc-A1*) and 13.4% (WT *vs. gpc-A1/gpc-B2*). Micronutrient concentrations in the mature grain for each genotype in UCD-2012 and TAU-2012 experiments are presented in Additional file 1: Table S1. Fe concentrations in the grain were significantly lower in both the *gpc-A1* (20.9% mean reduction) and *gpc-A1/gpc-B2* mutants (20.8% mean reduction) when compared to WT samples in both locations (Figure 2c). Zn grain concentrations were also lower for the same mutant genotypes in both locations, but the differences were significant only in the UCD-2012 experiment (Figure 2d). Interestingly, *gpc-A1* and *gpc-A1/gpc-B2* mutants also exhibited significantly higher grain K concentrations than in WT plants, with increases ranging between 18 and 33% (Additional file 1: Table S1). All GPC and micronutrient values are reported as the concentration within the grain, so are unaffected by the variation in TKW detected between genotypes.

Taken together, these results demonstrate that a knockout mutation of the *GPC1* gene alone is sufficient to delay the onset of senescence and to perturb the translocation of protein, Zn and Fe to the developing grain in tetraploid durum wheat under field conditions. The *gpc-B2* mutation had no significant effect on any of these traits, even in a genetic background with no functional *GPC1* genes.

### Evaluation of the mapping reference used for RNA-seq and overall characterization of loci expressed in each sample

To identify *GPC-*mediated transcriptional changes associated with the onset of senescence, we carried out an RNA-seq study focusing on three genotypes; WT and the two mutants that showed the largest differences in senescence in the previous field experiments, *gpc-A1* and *gpc-A1/gpc-B2*. None of the plants sampled at heading date (HD), 12 days after anthesis (DAA) or 22 DAA, showed signs of chlorophyll degradation in the flag leaves or yellowing of the peduncles (Additional file 1: Figure S3, a-c), confirming that the selected time points represent relatively early stages of the senescence process. Clear differences between genotypes were apparent five weeks later (60 DAA), when the WT plants showed more advanced symptoms of senescence than either of the two *gpc* mutants (Additional file 1: Figure S3, d-f). This result indicates that in this greenhouse experiment, the effects of the *GPC* genes were consistent with those observed in the field experiments described above (Figure 1a).

On average, 35 million trimmed RNA-seq reads were generated for each of the four replicates of each of the nine genotype/time point combinations included in this study (Additional file 1: Table S2, total 1.3 billion reads). Most of the reads (average 99.0%) were mapped to the reference genomic contigs generated by the International Wheat Genome Sequencing Consortium (IWGSC) using flow-sorted chromosomes arms of *T. aestivum* cv. Chinese Spring [48]. Since we were mapping transcripts of a tetraploid wheat cultivar, only the sequences from the A and B genome chromosome arms were used as a reference.

A large proportion of the trimmed reads (average 93.4%, Additional file 1: Table S2) mapped within the 139,828 previously defined transcribed genomic loci within this reference (see Methods), suggesting that these loci provide a good representation of the transcribed portion of the wheat genome. However, only 58.5% of these reads mapped to unique locations (Additional file 1: Table S2), most likely due to a combination of the high level of similarity shared by the coding regions of A and B homoeologs (average identity = 97.3%, standard deviation = 1.2%, [49]), and the short length of the reads used in this study (50 bp). Ambiguously mapped reads were excluded from the statistical analyses described below, resulting in an average of 20.4 M uniquely mapped reads per sample.

After excluding ambiguously mapped reads, only 80,168 of the genomic loci showed transcript coverage above the selected threshold for the statistical analyses (>3 reads for at least two biological replicates, within at least one genotype/time point pair, see Methods). The complete list of statistical analyses performed for these 80,168 loci is summarized in Additional file 2. Probability values for all four statistical tests are presented in this table so researchers can reanalyze the data using different statistical analyses and levels of stringency for specific sets of genes. Where available, this table also describes the high-confidence protein coding gene corresponding to each genomic locus, derived from the recent annotation of these wheat genomic contigs [48].

Principal component analysis (PCA) of the uniquely mapped reads at each time point showed limited clustering of the samples according to their genotype at HD (Additional file 1: Figure S4a), very clear groupings at 12 DAA (Additional file 1: Figure S4b), and intermediate clustering at 22 DAA (Additional file 1: Figure S4c). The reciprocal analysis, to distinguish samples according to time point within each genotype, showed that in all three genotypes, the HD samples were more clearly separated than the two later time points (Additional file 1: Figure S4, d-f). The clearer separation of both *gpc* mutants from the WT, and of *gpc-A1* from *gpc-A1/gpc-B2* at 12 DAA than at either HD or 22 DAA, suggests that both *GPC1* and *GPC2* genes have a major regulatory role at this early stage of senescence (12 DAA).

Following mapping, we confirmed the genotype of each sample by analyzing pileups of reads which mapped to the genomic loci corresponding to the *GPC-A1* and *GPC-B2* genes. The expected TILLING mutations (G561A = W114* for *gpc-A1* and G516A = W109* for *gpc-B2*) were confirmed in the expected mutant genotypes and were absent in all WT samples. All *GPC* genes showed a low number of mapped reads at HD, with significant increases at 12 DAA and 22 DAA (Additional file 1: figure S5). Approximately 3-4-fold more reads mapped to *GPC1* homoeologous genes than to the *GPC2* genes, a pattern which was consistent across all genotypes (Additional file 1: Figure S5).

We detected no significant differences in the expression profiles of *GPC-A1* and *GPC-B2* between WT and *gpc* mutant genotypes suggesting that the mutations in these genes did not affect the stability of the transcribed mRNAs, and that neither GPC-A1 nor GPC-B2 functional proteins exhibit a feedback regulatory mechanism on their own transcription (Additional file 1: Figure S5). However, at 22 DAA, *GPC-A2* expression was significantly lower in WT plants than in either *gpc-A1* (*P* = 0.024) or *gpc-A1/gpc-B2* (*P* = 0.004) mutants, suggesting that there may exist some *GPC*-mediated feedback mechanism on the regulation of *GPC-A2* transcript levels (Additional file 1: Figure S5).

### Identification of loci differentially expressed during monocarpic senescence in WT plants

**Figure 3:**
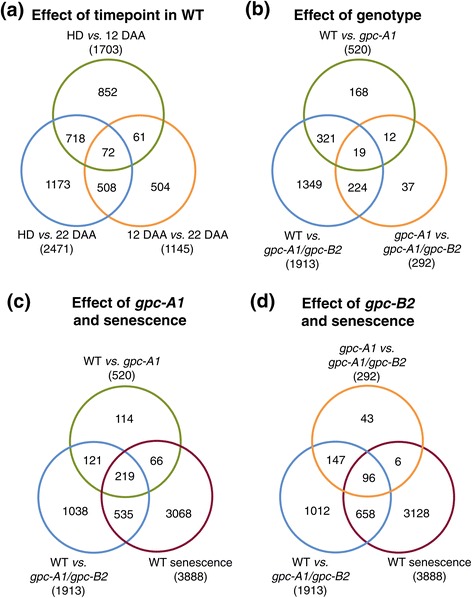
**Overlap of DE genes (a) Between time points in WT samples, (b) Between different** ***GPC*** **genotype comparisons, (c) Between** ***GPC-A1-*** **regulated loci and senescence regulated loci and (d) Between** ***GPC-B2-*** **regulated loci and senescence regulated loci.**

Applying stringent selection criteria (significant according to four different statistical tests, see Methods), we identified 3,888 contigs which were differentially expressed (DE) in at least one pairwise comparison among sampling times in the WT genotype (Figure 3a). As expected, the comparison between HD and 22 DAA showed the largest number of DE loci (2,471), followed by the comparison between HD and 12 DAA (1703). The comparison between 12 DAA and 22 DAA showed the lowest number of DE loci (1,145, Figure 3a).

Of the loci which were significantly DE in the WT plants between HD and 12 DAA, a larger proportion were upregulated (76.2%) than were downregulated (23.8%). The reverse was true for loci DE between 12 DAA and 22 DAA, when 30.2% of loci were upregulated and 69.8% were downregulated. This suggests that during the first 12 DAA different mechanisms required to actively prepare the plant for the upcoming senescence are upregulated, which is followed by the shutdown of many biological processes and the downregulation of a large number of genes.

We next determined whether any previously characterized senescence associated genes were also differentially expressed in our dataset. In a wheat microarray study, 165 annotated genes were identified which were differentially expressed during eight stages of senescence, ranging from anthesis to yellowing leaves [40]. We identified the corresponding genes within our dataset using BLAST (*P* ≤ 1e^−5^) and found that 26 (15.8%) were also significantly differentially expressed during senescence in the current study (Additional file 1: Table S3). This relatively low percent is not unexpected since our study covers only the early stages of senescence whereas the previous study covered a more extended period. A second microarray experiment in barley identified a set of genes differentially expressed between NILs divergent for a high-GPC genomic segment at 14 DAA and at 21 DAA [38]. In the leaves, 2,276 genes were upregulated in at least one of these time-points and 1,193 were downregulated. Among the upregulated genes, we identified 100 which were also significantly up-regulated during senescence, and of the down-regulated genes, 96 were also significantly down-regulated within our dataset, which used different statistical stringency criteria. The use of different technologies (microarray vs RNA-seq) and different species may also contribute to the different sets of differentially expressed genes detected in these studies. The genes regulated by senescence in both experiments are listed in Additional file 1: Table S4.

This study in tetraploid wheat supersedes our previous RNA-seq analysis in hexaploid wheat comparing the transcriptomes of WT and transgenic *GPC*-RNAi lines with reduced transcript levels of *GPC1* and *GPC2* at 12 DAA [39]. In the current study, we generated a greater number of reads, studied additional time-points, used targeted knockouts of individual *GPC* genes and had access to a more comprehensive wheat genome mapping reference. Among the differentially expressed genes common to both studies were three genes of biological interest selected for validation in the previous study [39].

### Identification of loci differentially expressed among *GPC* genotypes

We next identified loci which were DE between genotypes. The largest number of DE loci was detected between the WT and the double *gpc-A1/gpc-B2* mutants (1,913 loci), an expected result given that this comparison includes genes regulated by both *GPC-A1* and *GPC-B2* (Figure 3b). The comparison between the WT and the single *gpc-A1* mutant, expected to detect mainly *GPC-A1-*regulated genes, showed a much lower number of DE genes (520 loci) than the previous comparison. A total of 321 of these loci (62%, Figure 3b) were DE in both these comparisons and are designated hereafter as high-confidence *GPC-A1-*regulated genes. The third comparison, between the *gpc-A1* and *gpc-A1/gpc-B2* mutant genotypes, expected to detect mainly genes regulated by *GPC-B2*, yielded a lower number of DE loci (292). Most of these loci (224 = 77%, Figure 3b) were also DE in the comparison between the WT and the *gpc-A1/gpc-B2* double mutant and are designated hereafter as high-confidence *GPC-B2-*regulated genes. There were 19 loci which were DE in all three comparisons between genotypes, and these likely represent genes redundantly regulated by both *GPC-A1* and *GPC-B2* genes (Figure 3b). Similarly, the 1,349 loci DE only between the WT and double *gpc-A1/gpc-B2* mutants but not in the other two classes (Figure 3b), likely include loci that are redundantly regulated by both genes, but that show significant differences in expression only when mutations in both *GPC* paralogs are combined.

To determine how these differences between genotypes were distributed in time, we made pairwise comparisons between genotypes within each of the three time points. Since both *GPC1* and *GPC2* expression is relatively low at HD (Additional file 1 : Figure S5), we expected to find a small number of DE loci among *GPC* genotypes at this time point. Indeed, only ten genes were DE between WT and the *gpc-A1* single mutant, only six between WT and the *gpc-A1/gpc-B2* double mutant and 19 between the *gpc-A1* and *gpc-A1/gpc-B2* mutants at HD. Two loci were shared between the WT *vs. gpc-A1/gpc-B2* and *gpc-A1 vs. gpc-A1/gpc-B2* comparisons, suggesting they may potentially be regulated by *GPC-B2* and one gene was common to the WT *vs. gpc-A1* and WT *vs. gpc-A1/gpc-B2* comparisons, suggesting it may be regulated by *GPC-A1.* These results confirm that *GPC* genes have only a marginal effect on the wheat transcriptome at this developmental stage.

By contrast, the number of DE loci between genotypes was much greater at 12 DAA. Of the 520 loci DE between WT and the *gpc-A1* single mutant, 504 (96.9%) were DE at 12 DAA and only six (1.1%) at 22 DAA. Similarly, of the 1,913 loci DE between WT and the *gpc-A1/gpc-B2* double mutant 1,525 (79.7%) were DE at 12 DAA, whereas only 385 (20.1%) were DE at 22 DAA. Of the 292 DE genes in the comparison between the *gpc-A1* single mutant and the *gpc-A1/gpc-B2* double mutant, 239 were DE at 12 DAA, whereas only 38 genes were DE at 22 DAA. These results suggest that even though *GPC1* and *GPC2* expression continues to rise between 12 DAA and 22 DAA (Additional file 1 Figure S5), the major effect of both these genes on the regulation of downstream genes occurs at 12 DAA.

We next compared the two sets of high-confidence *GPC*-regulated loci with the senescence-regulated loci. A broad overlap was detected between *GPC-A1-*regulated and senescence-regulated loci, with 206 of the 321 (64.2%) high-confidence *GPC-A1-*regulated loci also DE during senescence (Figure 3c). By contrast, of the 224 high-confidence *GPC-B2-*regulated loci only 83 (37.1%) were also DE during senescence (Figure 3d). Surprisingly, 81% of the genes upregulated during the first 12 DAA in WT plants (1,054 genes) were no longer significant in the *gpc-A1* mutant. This observation highlights the critical role of *GPC1* in the activation of a large number of genes during the early stages of monocarpic senescence, possibly to prepare the plant for the upcoming senescence.

### Distribution of expression profiles among different genotypic classes

**Figure 4:**
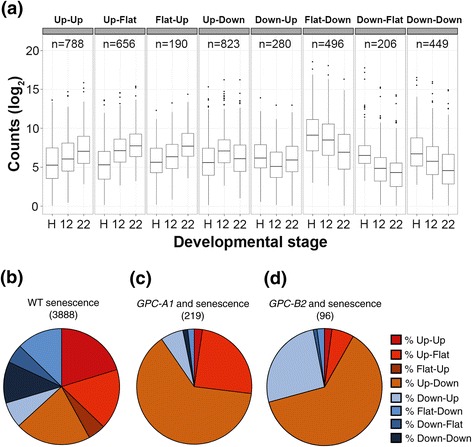
**Expression profiles during senescence. (a)** Boxplot of log_2_ normalized counts for WT samples over three time points during senescence, separated according to their expression profiles in 8 classes. Classes were defined based on the existence of significant (‘Up’ and ‘Down’) or non-significant differences (‘Flat’) between time point comparisons. **(b-d)** Proportion of expression classes among loci DE during senescence in **(b)** WT (3888 loci), **(c)** high-confidence *GPC-A1-*regulated loci (219 loci) and **(d)** high-confidence *GPC-B2-*regulated loci (96 loci). Loci included in C and D are based on the intersections of the three classes shown in Figure 3, c and d. H = Heading Date, 12 = 12 days after anthesis, 22 = 22 days after anthesis.

To further analyze the loci DE during senescence, we classified them into eight classes based on their upregulation (Up), downregulation (Down) or absence of significant differences (Flat) between HD and 12 DAA, and between 12 DAA to 22 DAA (Figure 4a). Loci which were not significantly DE in either of these comparisons, but were significantly up or downregulated between HD and 22 DAA were included in the ‘Up-Up’ and ‘Down-Down’ classes, respectively. When all 3,888 loci DE during senescence in WT plants were considered (Figure 4, a-b) all eight classes were well represented with slightly higher proportions in the three classes that include loci upregulated between HD and 12 DAA (‘Up-Down’: 21.2%, ‘Up-Up’: 20.3% and ‘Up-Flat’: 16.9%). A different picture emerged when, among the loci DE during senescence, we considered only the high-confidence *GPC-A1* (219) and *GPC-B2* (96) regulated genes*.* In both cases the ‘Up-Down’ class was dominant, representing 63.5% and 62.5% of the DE loci, respectively (Figure 4, c and d). However, a difference between these two groups was evident in the second most abundant class; ‘Up-Flat’ in the high-confidence *GPC-A1-*regulated genes (24.7%), and ‘Down-Up’ in the high-confidence *GPC-B2-*regulated genes (26.0%, Figure 4, c and d). In both groups, the remaining six classes represented less than 12% of the DE loci. These data indicate that while both genes have their greatest effect at 12 DAA, a partial differentiation exists of the loci and processes regulated by the *GPC-A1* and *GPC-B2* genes.

### Gene ontology analysis

We next used BLAST2GO to generate ‘Biological Process’ Gene Ontology (GO) terms for each locus to compare the proportions of different functional categories between loci up- and downregulated during senescence in WT and between high-confidence *GPC-A1-* and *GPC-B2-*regulated loci (Table 1). To simplify the description of these functional analyses, we first combined the eight functional categories from Figure 4a into four: upregulated loci (combining ‘Up-Up’, ‘Up-Flat’ and ‘Flat-Up’ categories), downregulated loci (combining ‘Down-Down’, ‘Down-Flat’ and ‘Flat-Down’ categories), ‘Up-Down’, and ‘Down-Up’.

**Table 1:** **Top significantly enriched ‘Biological Process’ GO terms among upregulated and downregulated genes during monocarpic senescence in wheat and in the 316 high-confidence** ***GPC-A1-*** **and 224** ***GPC-B2-*** **regulated genes**

	**Accession**	**Ontology**	**Annotated**	**Significant**	**Expected**	***P***
**Upregulated**	GO:0055114	Oxidation-reduction process	3168	171	87.5	2.60E-18
	GO:0055085	Transmembrane transport	1622	90	44.8	2.20E-10
	GO:0071577	Zinc ion transmembrane transport	20	8	0.55	3.10E-08
	GO:0034220	Ion transmembrane transport	369	31	10.19	4.90E-08
	GO:0006829	Zinc ion transport	24	8	0.66	1.60E-07
	GO:0043562	Cellular response to nitrogen levels	14	6	0.39	1.10E-06
	GO:0009064	Glutamine family amino acid metabolic process	65	11	1.8	1.50E-06
	GO:0006787	Porphyrin-containing compound catabolic process	76	11	2.1	7.40E-06
	GO:0033015	Tetrapyrrole catabolic process	76	11	2.1	7.40E-06
	GO:0051187	Cofactor catabolic process	76	11	2.1	7.40E-06
**Downregulated**	GO:0015979	Photosynthesis	502	123	11.06	<1e-30
	GO:0009765	Photosynthesis, light harvesting	76	47	1.67	<1e-30
	GO:0019684	Photosynthesis, light reaction	340	69	7.49	<1e-30
	GO:0006091	Generation of precursor metabolites and energy	774	78	17.06	1.70E-29
	GO:0033014	Tetrapyrrole biosynthetic process	183	30	4.03	9.60E-18
	GO:0015977	Carbon fixation	45	17	0.99	3.40E-17
	GO:0006779	Porphyrin-containing compound biosynthetic process	161	27	3.55	2.30E-16
	GO:0015995	Chlorophyll biosynthetic process	122	23	2.69	2.80E-15
	GO:0033013	Tetrapyrrole metabolic process	262	31	5.77	3.20E-14
	GO:0055114	Oxidation-reduction process	3168	134	69.81	5.50E-14
***GPC1-*** **regulated**	GO:0005385	Zinc ion transmembrane transporter activity	29	13	0.14	2.00E-23
	GO:0046915	Transition metal ion transmembrane transmembrane activity	68	13	0.32	7.80E-18
	GO:0072509	Divalent inorganic cation transmembrane activity	95	13	0.44	7.80E-16
	GO:0046873	Metal ion transmembrane transporter activity	317	15	1.48	3.00E-11
	GO:0022890	Inorganic cation transmembrane transport activity	472	15	2.21	7.20E-09
	GO:0022891	Substrate-specific transmembrane transport	1016	20	4.76	6.60E-08
	GO:0015075	Ion transmembrane transporter activity	911	18	4.26	3.00E-07
	GO:0022892	Substrate-specific transporter activity	1132	20	5.3	3.70E-07
	GO:0008324	Cation transmembrane transporter activity	647	15	3.03	4.30E-07
	GO:0005215	Transporter activity	1989	26	9.31	1.90E-06
***GPC2-*** **regulated**	GO:0009834	Secondary cell wall biogenesis	17	2	0.03	0.00041
	GO:0009832	Plant-type cell wall biogenesis	52	2	0.09	0.00382
	GO:0007017	Microtubule-based process	379	4	0.67	0.00446
	GO:0006812	Cation transport	948	6	1.67	0.00623
	GO:0071669	Plant-type cell wall organization or biogenesis	69	2	0.12	0.00664
	GO:0006811	Ion transport	1285	7	2.27	0.00701
	GO:0007029	Endoplasmic reticulum organization	5	1	0.01	0.00879
	GO:0015801	Aromatic amino acid transport	5	1	0.01	0.00879

Among loci upregulated during senescence, we observed enrichment in transport functions and catabolism of photosynthetic proteins. Four of the top five most significantly enriched GO terms included those related to transmembrane transporter function (Table 1). By contrast, loci downregulated during senescence were enriched in functions related to biosynthetic processes, especially photosynthesis (Table 1). These results, together with the previous observation that upregulated loci were more abundant between WT and 12 DAA (76.2%) and downregulated loci were more abundant between 12 and 22 DAA (69.8%), are indicative of the early activation of catabolic enzymes and transport systems followed by the downregulation of growth promoting processes in the leaves during these two early stages of senescence.

GO term analysis among the 321 high-confidence *GPC-A1-*regulated genes showed a significant enrichment of categories similar to the patterns observed for loci upregulated during senescence, with the ten most significantly enriched terms all relating to transporter activity (Table 1). Although transporter functions were also enriched among the 224 high-confidence *GPC-B2-*regulated genes, several unrelated terms were also enriched in this class but not in the *GPC-A1-*regulated class, including genes with putative roles in cell wall biogenesis and microtubule organization.

The closer similarity in GO term enrichment between senescence-regulated loci and *GPC-A1-*regulated genes than with *GPC-B2-*regulated genes is consistent with the greater overlap between senescence-regulated and *GPC-*regulated loci (64.2% overlap for *GPC-A1 vs.* 37.1% overlap for *GPC-B2*, Figure 3, c and d) and with the relatively stronger effect of the *gpc-A1* mutation on senescence and nutrient transport relative to the *gpc-B2* mutation (Figure 1, a-d). Taken together, these results suggest that *GPC-A1* plays a more important role than *GPC-B2* in the regulation of genes controlling the early stages of monocarpic senescence in wheat.

### Identification and expression analysis of wheat transporter genes

To categorize the wheat transporters upregulated during senescence and to determine the role of *GPC1* in their regulation, we identified specific wheat homologues of Fe and Zn transporters previously characterized in other plant species and determined their expression profiles both among different time points during senescence and between *GPC* genotypes.

#### Chloroplastic transporters

Among genes previously known to be involved in the reduction-based import of Fe into the chloroplasts, we identified two *FRO* genes in *Triticum aestivum* (*Ta*), one of which, *TaFRO1*, was highly expressed at HD and significantly downregulated during senescence in WT plants (Table 2). By comparison, *TaFRO2* expression was lower, and although its expression also fell during senescence, differences between time points were not significant. Neither gene was significantly DE among genotypes.

**Table 2:** **Wheat transporters and their expression during senescence**

**Transporter**				WT counts*			Differential expression**		
**Rice**	**Wheat**	**Chromosome**	**IWGSC ID**	**HD**	**12D**	**22D**	**Senescence**	***gpc-A1***	***gpc-A1/B2***
**Chloroplastic transporters**									
*OsFRO1*	*TaFRO1*	2AL	Traes_2AL_2A274FDB8	40,148	22,892	16,515			
		2BL	Traes_2BL_C7CDCB39A	48,913	30,272	13,838	⬇		
*OsFRO2*	*TaFRO2*	2AL	Traes_2AL_7E818894E	3	1	6			
		2BL	Traes_2BL_E11AA2D03	49	8	9			
*OsHMA1*	*TaHMA1*	7AL	Traes_7AL_84D5BAE85	1,143	1,256	1,249			
		7BL	Traes_7BL_041308E74	674	614	600			
	*TaHMA1-like*	5AL	Traes_5AL_C89EEBE50	1	2	1			
		5BL*‡*	Traes_5BL_F83C809F0	74	78	116			
*OsHMA2*	*TaHMA2*	7AL	Traes_7AL_8304348B7	125	328	1,079	⬆		
		7BL	Traes_7BL_C46BC291C/ Traes_7BL_8C24C1025	185	356	1,205	⬆		
	*TaHMA2-like*	7AL	Traes_7AL_6AE850114	13	75	188	⬆	⬆	⬆
		7BL	Traes_7BL_0CF58CF4E	2	13	17	⬆		
*OsHMA3*	*TaHMA3*	5BL	Traes_5BL_D6C3DC326	-	-	-			
**Vacuolar transporters**									
*OsVIT1*	*TaVIT1*	2AL	Traes_2AL_A3A25F40E	59	44	36			
		2BL	Traes_2BL_54954138A	74	61	54			
*OsVIT2*	*TaVIT2*	5BL	Traes_5BL_7CE3EDE29	1,938	566	450			
*OsNRAMP1*	*TaNRAMP1*	7AL	Traes_7AL_76159C6DA	-	-	-			
		7BL	Traes_7BL_03741F576	3	6	1			
*OsNRAMP2*	*TaNRAMP2*	4AS	Traes_4AS_BBF51CA2E	437	927	3,607	⬆		
		4BL	Traes_4BL_C6A3F5C8A	637	743	2,252			
*OsNRAMP3*	*TaNRAMP3*	7AL	Traes_7AL_08B2A7BB2	640	704	469			
		7BL	Traes_7BL_CA6B7C9E6	551	571	352			
*OsNRAMP4*	*TaNRAMP4*	6AS	Traes_6AS_B9B4AD633	1	1	1			
		6BS	N/A	3	5	5			
*OsNRAMP5*	*TaNRAMP5*	4AS	Traes_4AS_5D4904831	6	2	2			
		4BL	Traes_4BL_04B01EA0C/ Traes_4BL_CFD804098	4	2	2			
None	*TaNRAMP6*	3AS	Traes_3AS_538630B00	-	-	-			
		3B	Traes_3B_73F0469A5	10	9	19			
*OsNRAMP7*	*TaNRAMP7*	5AS	Traes_5AS_213BE4D84	215	176	151			
		5BS	Traes_5BS_DE1CD2DA4	127	88	103			
None	*TaNRAMP8*	4AL	Traes_4AL_2E796609C	-	-	-			
		4BS	Traes_4BS_9337E9B2F	-	-	-			
*OsZIFL1*	*TaZIFL1*	3AS	Traes_3AS_C25151458	378	73	19	⬇		
		3B	Traes_3B_BD45F6269/ Traes_3B_45F864939	249	45	5	⬇		
*OsZIFL1*	*TaZIFL1-like1*	3AS	Traes_3AS_02DE247DA	125	136	84			
		3B	Traes_3B_92383792E/ Traes_3B_B76607C0E	70	76	59			
*OsZIFL2*	*TaZIFL2*	5BL	Traes_5BL_A0B9DE62E	1	2	2			
*OsZIFL2*	*TaZIFL2-like1*	3AS	Traes_3AS_E59FB52EC	8	31	32	⬆		
		3B	Traes_3B_EDFDD5A12	167	305	383			
None	*TaZIFL3*	4AL	Traes_4AL_4231650FC/ Traes_4AL_470869233	183	218	171			
		4BS	Traes_4BS_1DCF82CB7	525	1,019	743	⬆		
None	*TaZIFL7*	5AL	Traes_5AL_37BFFFD9E	397	392	249		⬆	⬆
		5BL	Traes_5BL_6E4AE0146	130	108	48			
None	*TaZIFL8*	4AL	Traes_4AL_5C7A4DA54	21	19	13			
		4BS	Traes_4BS_44732F50F	141	112	160			
None	*TaZIFL9*	5AL	Traes_5AL_0599F7BC5	-	-	-			
		5BL	N/A	-	-	-			
**Plasma membrane transporters**									
*OsYSL1*	*TaYSL1*	3AS	Traes_3AS_FB4110335	10	7	8			
		3B	Traes_3B_17BC3E1E2	1	1	1			
*OsYSL2*	*TaYSL2*	6AL	Traes_6AL_850660AC3	56	35	53			
		6BL	Traes_6BL_3DD0BA741	51	61	62			
*OsYSL6*	*TaYSL6*	2AL	Traes_2AL_7B0F93F84	364	526	986	⬆		
		2BL	Traes_2BL_0CBCC13AD	68	101	203			
*OsYSL9*	*TaYSL9*	2AL	Traes_2AL_CFCA01C76	725	995	1,517	⬆		
		2BL	N/A	15	18	28			
*OsYSL10*	*TaYSL10*	6AL	Traes_6AL_5642D5B44	42	46	35			
*OsYSL11*	*TaYSL11*	2AL	Traes_2AL_377C8CDEA	0	0	0			
		2BL	Traes_2BL_68E0CA743	3	5	3			
*OsYSL12*	*TaYSL12*	2AL*‡*	Traes_2AL_CC6133527	554	701	1,389			
		2BL	Traes_2BL_2BE05F104	437	563	454			⬇
*OsYSL13*	*TaYSL13*	2AL	Traes_2AL_F707FF2C3.3	14	20	75			
		2BL	Traes_2BL_A14EA5AE4	1	1	3			
	*TaYSL13-like*	2BL	Traes_2BL_A14EA5AE4	7	19	85			
*OsYSL14*	*TaYSL14*	6AL	Traes_6AL_7FB45D4DE	814	894	733			
		6BL	Traes_6BL_7FFC46B84	500	628	493			
*OsYSL15*	*TaYSL15*	6AL	Traes_6AL_E36FCEF64	523	486	969			
		6BL	Traes_6BL_D65EC1432	88	95	199			
	*TaYSL15-like*	1AL	Traes_1AL_C6A0E255E	4	7	9			
		1BL	Traes_1BL_CA93E6359	2	2	2			
*OsYSL16*	*TaYSL16*	2BL	Traes_2BL_4A1181B731	2,736	2,361	2,011			
*None*	*TaYSL18*	2AL	Traes_2AL_2F91AF932	225	144	84	⬇		
		2BL	Traes_2BL_6C5206B6D	531	499	510			
*OsIRT1*	*TaIRT-like1*	4AL	Traes_4AL_9D79BE8FB	5	7	10			
		4BS	Traes_4BS_6527BBD54	8	6	7			
*OsIRT2*	*TaIRT-like 2*	4AL	Traes_4AL_9F6B106F3	9	21	30			⬆
*OsZIP10*	*TaZIP10*	7AL	Traes_7AL_A13A246B4	333	596	531		⬆	⬆
		7BL	Traes_7BL_E5CFC3DCE/ Traes_7BL_5C965DB64	267	531	482		⬆	⬆
*OsZIP10*	*TaZIP10-like1*	7AL	Traes_7AL_F1D611563/ Traes_7AL_893DEB3EB	7	229	279	⬆	⬆	⬆
		7BL	Traes_7BL_12C63350C	5	654	647	⬆	⬆	⬆
*OsZIP8*	*TaZIP13-like1*	6BS	Traes_6BS_7D630200B	2	2	2			
	*TaZIP13-like2*	2AS	Traes_2AS_4CA7607E5	277	4,469	3,207	⬆	⬆	⬆
		2BS	Traes_2BS_9D8F265EC	79	916	787	⬆	⬆	⬆
	*TaZIP13-like3*	2AL	Traes_2AL_BE05B34FF	125	129	135			
		2BL	Traes_2BL_A1BCBD2BE	102	1,613	1,466	⬆	⬆	⬆
*OsZIP1*	*TaZIP1†*	3AL	Traes_3AL_DC3D5F65E	2	1	4			
		3B	Traes_3B_3C18D89F8	3	4	8			
*OsZIP2*	*TaZIP2†*	5BL	N/A	17	9	9			
*OsZIP2*	*TaZIP2-like*	6AS	Traes_6AS_75C6428051	-	-	-			
		6BS	N/A	73	65	34			
*OsZIP3*	*TaZIP3†*	2AL	Traes_2AL_3983FD077	21	115	138	⬆	⬆	⬆
		2BL	Traes_2BL_23F1C8743	61	130	291		⬆	⬆
*OsZIP5*	*TaZIP5*	4AS	Traes_4AS_F5F7D2A8D	0	1	3			
		4BL	Traes_4BL_68691F1FC	10	76	176		⬆	⬆
*OsZIP6*	*TaZIP6†*	1AS	Traes_1AS_A6EF18CC1	527	488	439			
		1BS	Traes_1BS_B734EDEA7	413	532	478			
*OsZIP7*	*TaZIP7†*	1AS	Traes_1AS_EC8891094/ Traes_1AS_50323685E	216	606	880	⬆	⬆	⬆
		1BS	Traes_1BS_D68F0BED6	317	1,194	1,566	⬆	⬆	⬆
*OsZIP11*	*TaZIP11*	1AS	Traes_1AS_7DC2CB902	378	357	519			
		1BS	N/A	193	186	281			
*OsZIP13*	*TaZIP15*	6AS	Traes_6AS_4E1D574BC	322	354	332			
		6BS	Traes_6BS_147BF2D07	726	678	513			
*OsZIP14*	*TaZIP14†*	3AS	Traes_3AS_F46E02204/ Traes_3AS_15A221AD0	97	77	103			
		3B	Traes_3B_B7D3B69FD	229	192	213			
*OsZIP16*	*TaZIP16*	7AL	Traes_7AL_DFE86911E	15	14	9			
		7BL	Traes_7BL_2637B2942/ Traes_7BL_EB231D8FF	12	13	6			
**PS biosynthesis genes**									
*OsNAS1*	*TaNAS1*	2AS	Traes_2AS_D50EEDA84	2	5	4			
		2BS	N/A	121	117	117			
*OsNAS3*	*TaNAS3*	2AS	Traes_2AS_DEDC612AE/ Traes_2AS_452FED53F	2,056	2,694	4,495			⬆
		2BS	Traes_2BS_CB79BAFB1	5,362	5,109	7,789			⬆
*OsNAAT1*	*TaNAAT1*	1AL	Traes_1AL_9D6B86169	47	88	102		⬆	⬆
*OsNAAT1*	*TaNAAT2*	1AL	Traes_1AL_BCD7C5B8B	127	280	269	⬆	⬆	⬆
		1BL	Traes_1BL_D8276D3DB	188	453	602		⬆	⬆
*OsDMAS1*	*TaDMAS1*	4AS	Traes_4AS_887399584	27	26	17			
		4BL	N/A	1222	883	1134			

† Source: Tiong et al. [21]. All *NRAMP* genes from Borrill et al. [7]. *TaDMAS* from Bashir et al. [26]. *‡* Predicted non-functional protein.

*Counts are normalized values of reads uniquely mapped to the genomic loci corresponding to each wheat transporter gene. **Arrows indicate whether the gene was significantly upregulated (⬆) or downregulated (⬇) during senescence in WT plants or in WT *vs. gpc-A1* or WT *vs. gpc-A1/gpc-B2* comparisons (⬆ = significantly higher in WT).

Among genes previously known to promote the export of nutrients from the chloroplast to the cytoplasm, we identified five *T. aestivum* members of the Zn/Co/Cd/Pb-transporting class of *HMA* genes (see phylogeny in Additional file 1: Figure S6). Two of these genes, *TaHMA2* and *TaHMA2-like*, which showed the highest similarity to *OsHMA2* (Additional file 1: Figure S6), were significantly upregulated during senescence, both showing >6-fold increases in expression between HD and 22 DAA (Table 2). Furthermore, *TaHMA2-like* expression was significantly reduced in both *gpc* mutants, implicating a role for *GPC* in its regulation. Two other genes, *TaHMA1* and *TaHMA-like1* which are both similar to *OsHMA1* (Additional file 1: Figure S6), were not DE during senescence and a third, *TaHMA3*, was not detected at any time point in this study.

#### Vacuolar transporters

Two *VIT* transporters, which promote Fe and Zn import in to the vacuole, were previously characterized in rice [13]. Both of the corresponding wheat homologues of these genes were downregulated ~4-fold during senescence, but these differences were not significant according to our stringent differential expression criteria (Table 2). Furthermore, neither gene was DE in either of the *gpc* mutant genotypes (Table 2).

Eight wheat *ZIFL* genes, thought to promote vacuolar sequestration of Zn [14], were identified and annotated in this study (see phylogeny in Additional file 1: Figure S7). Two *TaZIFL* genes (*TaZIFL2* and *TaZIFL9*) were expressed at negligible levels in all time points included in this study and were excluded from further analyses (Table 2). Among the six *TaZIFL* genes which showed higher levels of expression during senescence, *TaZIFL2-like1* and *TaZIFL3* were significantly upregulated during senescence while *TaZIFL1* was significantly downregulated. Interestingly, although it was not upregulated during senescence, *TaZIFL7* expression was significantly higher in WT plants than in both *gpc* mutants (Table 2).

Among the genes known to promote Fe export from the vacuole to the cytoplasm, eight *NRAMP* genes were recently described in wheat [7]. Five of these genes showed very low levels of expression in flag leaves during the time points included in our study, suggesting that they may play more important roles during other developmental stages or in other tissues. Of the three *NRAMP* genes with higher expression levels during senescence, *TaNRAMP3* and *TaNRAMP7* both exhibited stable expression, but *TaNRAMP2* was significantly upregulated, showing a ~5-fold increase in expression between HD and 22 DAA (Table 2). No significant differences among genotypes were detected for any of the *NRAMP* genes.

#### Plasma-membrane transporters

After being transported into the cytoplasm, Zn and Fe must be loaded into the phloem for their transport to different sink tissues, including the grain. In rice and barley, the *YSL* and *ZIP* gene families appear to play a prominent role in this process.

We identified a total of 14 *YSL* genes within available wheat databases (see phylogeny in Fig S8), but one of these genes is likely a pseudogene (Table 2). Among the functional *YSL* genes, *TaYSL6* and *TaYSL9* were significantly upregulated during senescence and *TaYSL18* was significantly downregulated (Table 2). Although not DE during senescence, *TaYSL12* expression was significantly reduced in the *gpc-A1/gpc-B2* mutant compared to the WT.

The largest transporter gene family described in this study is the *ZIP* family, with a total of 19 wheat genes identified (see phylogeny in Additional file 1: Figure S9), including seven which had been described previously [21]. This family also includes the Iron Regulated Transporter (IRT) genes, which share high similarity to the ZIPs. One gene (*TaZIP13*) was absent from the genomic reference so was excluded from the analysis, but five of the remaining 18 *TaZIP* genes were significantly upregulated during senescence (Table 2). Some of these genes showed very large increases in expression between time points. For example, *TaZIP3* was upregulated 5-fold and *TaZIP5* 8-fold between HD and 12 DAA (Table 2). Strikingly, the expression of all five of these upregulated genes, as well as *TaZIP10* and *TaZIP5*, was significantly higher in WT plants than in either *gpc* mutant genotype. Additionally, *TaIRT2* expression was significantly lower in the *gpc-A1/gpc-B2* mutant than in the WT. These results strongly implicate a role for *GPC1* in the regulation of the ZIP family of transporters during senescence (Table 2).

#### Phytosiderophore biosynthesis genes

Since the association of Zn and Fe with PS chelating ligands facilitates their transport through the phloem, we searched for wheat homologs of genes encoding enzymes acting in the PS biosynthetic pathway (Figure 1). Searches of available wheat genomic databases yielded two *TaNAS*, two *TaNAAT* and one *TaDMAS* genes.

Expression of *TaNAS3* more than doubled between 12 DAA and 22 DAA, (although this difference was not significant according to our criteria) and was significantly reduced in *gpc-A1/gpc-B2* mutant compared to the WT (Table 2). In contrast, *TaNAS1* was expressed at much lower levels and did not vary during senescence or among genotypes (Table 2).

Both of the identified wheat *NAAT* genes were upregulated during senescence, although only for *TaNAAT2* was this significant (Table 2). Interestingly, *TaNAAT2* was upregulated at an earlier stage than *TaNAS3*, since its expression doubled between HD and 12 DAA and remained stable thereafter (Table 2). The expression of both *TaNAAT* genes was significantly lower in both *gpc* mutant genotypes, suggesting a role for *GPC* in the regulation of this class of gene. The third PS biosynthesis gene, *TaDMAS* was not DE at any stage of senescence or in any of the genotype comparisons (Table 2).

As a technical control, we developed qRT-PCR assays for six transporter genes which were significantly DE between WT and *gpc* mutants. For all six genes, we obtained results that were consistent with the expression profiles determined by RNA-seq. Results from both analyses are presented side by side in Additional file 1: Figure S10.

To look for additional transporters, we further explored the group of 1,054 genes that were significantly upregulated between HD and 12 DAA in the WT plants but not in the *gpc-A1* mutants. This dataset included 33 genes with annotated transporter function which were also significantly different among genotypes (*P* < 0.05), 11 of which were members of the characterized transporter families described above (Additional file 1: Table S5). The remaining 22 genes included members of other transporter families, including one potassium transporter (AKT2), two sulfate transporters, one ABC transporter and a gene encoding a ferritin protein, involved in Fe storage (Additional file 1: Table S5). The differential regulation of the potassium and sulfate transporters is particularly interesting given the significant differences in K and S concentrations in the grain detected between WT and both *gpc-A1* and *gpc-A1/gpc-B2* mutants (Additional file 1: Table S1).

Taken together, our results suggest that the onset of monocarpic senescence in wheat is associated with broad transcriptional changes involved in nutrient remobilization. These processes included the export of Zn and Fe from chloroplasts and vacuoles (upregulation of *NRAMP* and *HMA* and downregulation of *FRO*, *VIT* and one *ZIFL* gene), upregulation of trans-membrane transporter genes responsible for loading nutrients into the phloem (*ZIP* and *YSL*), and upregulation of PS biosynthesis genes (*NAS*, *NAAT*) to facilitate transport of these nutrients through the phloem. Among these changes, the *GPC* genes seem to play a limited role in the regulation of vacuolar and chloroplastic transporter genes, but have a clear role in the upregulation of both PS biosynthesis genes and transmembrane transporters, with a particularly prominent role in regulating members of the *ZIP* gene family.

## Discussion

In annual grasses, senescing leaves are an important source of Zn and Fe for the developing grain. When the transport mechanisms between these tissues are disrupted, as in the *gpc* mutants and *GPC-*RNAi transgenic plants described in this and previous studies [5,44,46], concentrations of Zn and Fe in the grain are significantly reduced. We used RNA-seq to characterize the overall transcriptional changes in senescing flag leaves in WT and *gpc* mutant plants. We identified several Zn and Fe transporter genes activated during these early stages of senescence and describe their regulation by the *GPC* genes.

### Applying RNA-seq to polyploidy wheat

One challenge for genomic studies in polyploid species is the difficulty in distinguishing highly similar homoeologous genomes (~97% identical between A and B wheat genomes within protein-coding regions). Although different approaches to separate homoeologous sequences have been applied (e.g. Krasileva et al. [49]), it remains difficult to fully resolve chimeric assemblies. We overcame this problem in the current study by using the recently-released genomic draft sequence of wheat chromosome arms from the IWGSC as our RNA-seq mapping reference [48]. To generate the genomic draft sequence, wheat chromosome arms were first separated by flow cytometry, so each arm was sequenced and assembled separately, resulting in homoeolog-specific reference sequences.

At the time of our analysis, this genomic reference lacked any gene annotation so we first identified genomic ranges, which are defined by one or more overlapping transcripts (described in Methods). A large proportion of our reads mapped to these expressed loci (>93%), indicating that they include a good representation of the expressed portion of the wheat genome. Recently, the IWGSC annotated 65,776 high-confidence protein-coding genes in the A and B chromosome arms [48], 48,657 (74.0%) of which overlapped with loci identified in our study. The corresponding IWGSC loci and gene names for each matching locus are provided in Additional File 1. A small number of sequencing reads (average 209,660 reads per sample) mapped within genomic ranges defined by the 17,119 IWGSC loci not identified in our annotation, and were not included in the current study.

The use of a homoeolog-specific genomic reference instead of a transcriptome reduced mapping ambiguity in two ways; firstly by eliminating chimeric assemblies of similar homoeologous sequences, and secondly by collapsing multiple transcribed variant sequences into a single genomic locus in the reference, thus eliminating redundancy and increasing mapping specificity. This approach combines the expression of alternative splicing forms, which we consider appropriate for this initial study. A relatively high proportion of these reads were mapped uniquely (58.5%), and only these reads were used for our differential expression analyses, thus maximizing the accuracy of these analyses.

### The role of *GPC1* and *GPC2* during monocarpic senescence

Previous studies have demonstrated that transgenic plants expressing an RNAi construct targeting all copies of *GPC1* and *GPC2* exhibit a significant delay in senescence and reduced levels of Zn, Fe and protein in the grain, due to a disruption in their transport [5,44]. In the current study, we also detected a marginally significant reduction in TKW and in dry spike weight during grain filling associated with the *gpc1* mutant genotype. Interestingly, these differences were evident even from the early stages of senescence (35 DAA, Additional File 1: Figure S2b) suggesting that the *GPC* genes may also affect the rate of grain filling. Although the differences in spike weight between genotypes decreased with time, they were still significant at the end of the grain filling period (Additional file 1: Figure S2a). The high spring temperatures characteristic of the Mediterranean environments used in our studies, may have contributed to the lower kernel weight of the *gpc* mutant lines that matured during periods of higher temperature than the WT lines.

It was previously unknown whether *GPC* genes regulated the induction of the overall senescence process, or whether they regulated just a subset of the genes differentially regulated during this developmental stage. Also unclear was the time within the senescence process when the *GPC* genes have their strongest effect, or the extent of functional overlap between *GPC1* and *GPC2* paralogs. Results from our study provide insights into all three of these questions.

#### Comparing senescence- and GPC-regulated genes

To answer the first question, we investigated the overlap between senescence and *GPC* regulated genes. Of the 3,888 loci DE during senescence in WT plants, only 21.2% also showed significant differences in expression between *GPC* genotypes (Figure 3, c and d). In addition, within the senescence-regulated genes the subset regulated by the *GPC* genes showed different proportions of expression categories compared to the complete senescence set (Figure 4, b-d). Whereas more than 60% of *GPC-*regulated genes in this set exhibited an ‘Up-Down’ expression profile, the proportion in all senescence-regulated genes was 20%. Conversely, the proportion of genes exhibiting an ‘Up-Up’ expression profile and those falling into any of the downregulated classes were 9–10 fold more abundant among senescence-regulated genes than the subset regulated by *GPC* genes (Figure 4, b-d).

These results support the hypothesis that the *GPC* genes regulate a specific subset of genes during monocarpic senescence rather than triggering the overall transcriptional regulatory cascade associated with this developmental stage. The disruption of the regulation of this subset of senescence-regulated genes in the *gpc* mutants is likely sufficient to generate bottlenecks in the senescence process, as demonstrated by the overall delay in senescence observed in these mutant genotypes (Figure 2).

#### GPC-regulated genes at different time points

In a PCA analysis based on the expression of all genes, the differences between WT and *gpc* mutant genotypes were much clearer at 12 DAA than at either HD or 22 DAA (Additional file 1: Figure S4, a-c). This is consistent with our finding that the majority of *GPC-*regulated genes (76.1%) were detected at 12 DAA. These results suggest that, despite the continued increase in *GPC* expression between 12 DAA and 22 DAA (Additional file 1: Figure S5), most of the regulatory effects of the *GPC* genes on the DE of downstream genes occur within the first 12 DAA.

#### Comparison between GPC1 and GPC2 regulated genes

The paralogous genes *GPC1* and *GPC2* share 91% similarity at the DNA level and have almost identical expression profiles during the early stages of senescence (Additional file 1: Figure S5). Therefore, some functional overlap between these genes was expected. Among the three pairwise comparisons among genotypes (Figure 3b), the largest category includes the 1,349 DE genes detected between the WT and the *gpc-A1/gpc-B2* mutant, that were not among the *GPC-A1-*specific (WT *vs. gpc-A1*) or *GPC-B2-*specific (*gpc-A1/gpc-B2 vs. gpc-A1*) DE genes. This category most likely includes genes that are redundantly regulated by *GPC-A1* and *GPC-B2*, but that are significant only when both genes are absent. Combining these 1,349 genes with the 31 which are DE in both *GPC-A1-*specific and *GPC-B2-*specific comparisons (Figure 3b), we conclude that approximately two-thirds of the *GPC-*regulated genes (64.8%) showed some level of redundancy in their regulation by *GPC-A1* and *GPC-B2*. This result parallels the similar proportion of ‘Up-Down’ regulated genes among the high confidence *GPC-A1-* and *GPC-B2-*regulated genes (63.5% and 62.5%, respectively, Figure 4, c and d).

However, the remaining one-third of the loci DE among genotypes were regulated either by *GPC-A1* (489) or by *GPC-B2* (261), suggestive of some level of functional divergence. This was apparent in the differences between classes in expression profiles during senescence. Both sets of genes showed a strong enrichment for ‘Up-Down’ regulated genes, but the second most abundant category was ‘Up-Flat’ in the *GPC-A1-*regulated genes (24.7%) and ‘Down-Up’ in the *GPC-B2-*regulated genes (26.0%, Figure 4, c and d). These results suggest that *GPC-A1* acts principally to upregulate genes during the early stage of senescence (WT to 12 DAA), whereas *GPC-B2*, in addition to its role in the upregulation of a number of genes, also targets a subset of genes for downregulation during the same period.

Distinctions between *GPC-A1-* and *GPC-B2-*regulated genes were also apparent in their putative functions identified in the GO analysis and in their respective overlap with senescence-regulated genes. The proportion of *GPC-A1-*regulated genes that were also regulated by senescence (64.2%) was almost double the corresponding proportion of *GPC-B2*-regulated genes (37.1%, Figure 3, c and d). Moreover, putative functions of the senescence regulated genes were more similar to the functions of *GPC-A1-*regulated genes than to those regulated by *GPC-B2*. Whereas both senescence-regulated and *GPC-A1-*regulated genes were significantly enriched for transporter function (see section below), such enrichment was less evident among the *GPC-B2-*regulated genes. Instead, this class was enriched for genes with putative roles in plant cell wall biogenesis, microtubule organization and other processes distinct from those found in the senescence-regulated genes.

These results are consistent with the stronger effect of *gpc-A1* knockout mutants on senescence and nutrient translocation profiles in the current study (Figure 1, a-d) and with the strong effect seen on these phenotypes in *gpc1-*null mutants in hexaploid wheat [46]. One caveat of this comparison is that while the *gpc-A1* mutation in the tetraploid variety ‘Kronos’ represents a true *gpc1-*null allele (because of the natural non-functional mutation in *GPC-B1* in this variety), the *gpc-B2* mutant likely alters the dosage of *GPC2* but does not result in a *gpc2-*null mutant (because of the presence of an intact and expressed copy of *GPC-A2*). No *gpc-A2* truncation mutant was found in our current tetraploid TILLING population, but we are currently transferring a *gpc-A2* premature stop codon mutant found in our hexaploid wheat TILLING population [50] into Kronos. The lack of a *gpc-A2* truncation mutant in this study does not affect the interpretation of the *GPC-B2* regulated genes, but may have resulted in an underestimation of the number of genes regulated by *GPC2.*

Despite the high sequence similarity of *GPC1* and *GPC2* and their common expression profiles in the wheat flag leaf during monocarpic senescence, these genes appear to have diverged to regulate different sets of downstream targets. The closest rice ortholog to the wheat *GPC* genes is *Os07g37920* which maps to a region of the genome collinear to *GPC2* [51]. This suggests that *GPC2* is the ancestral gene and that *GPC1* originated from a duplication event specific to the wheat lineage [51]. However, the downregulation of *Os07g37920* by RNAi, or its overexpression in transgenic rice plants, did not affect the rate of senescence. The only difference observed in *Os07g37920-*RNAi rice plants was male sterility caused by the inability of the anther to dehisce and release pollen [51]. These results suggest that the specialization of *GPC1* on the regulation of transporters during senescence, and its stronger effect on the rate of senescence, are likely derived characteristics acquired by *GPC1* after its duplication to its non-orthologous location on the short arm of homoeologous group 6 chromosomes.

A recent study showed that the rice gene OsNAP, a close paralogue of Os07g37920 and of wheat GPC1, has a strong effect on senescence ([52], PNAS 111: 10013–10018).

In summary, the results described in this section indicate that the *GPC* genes regulate a subset of senescence-regulated genes that are mainly upregulated during the early stages of monocarpic senescence (first 12 DAA). Although most *GPC*-regulated genes are affected by both paralogs, *GPC1* seems to play a stronger role than *GPC2* in the regulation of a subset of genes involved in senescence that includes enrichment for transporter gene function. However, in the absence of a complete knockout of *GPC2,* we cannot fully determine the role of *GPC2* in monocarpic senescence. The importance of *GPC1* in the regulation of senescence is highlighted by the finding that among the 1,298 genes upregulated in the WT between HD and 12 DAA 1,054 were not upregulated in the *gpc-A1* mutant.

### Effect of *GPC* genes on previously characterized genes involved in transport

To complement the top-down analyses discussed above, we also characterized the effect of *GPC* and senescence on the expression of nine gene families previously shown to play important roles in nutrient remobilization in other plant species. The discussion of these genes is organized according to their involvement in different transport processes.

#### Chloroplast to cytoplasm

Our results demonstrate that the early stages of monocarpic senescence in the flag leaf are associated with a large-scale downregulation of genes involved in photosynthetic processes (Table 1). Therefore, the downregulation of *TaFRO1*, which is likely involved in the import of Fe into the chloroplast is not surprising. Studies in model species have demonstrated that some members of the FRO family localize to the chloroplast membrane and act in the reduction-based import of nutrients into the chloroplast [9,10]. In rice, *OsFRO1* transcript levels are negatively correlated with Zn and Fe levels in the rice grain [53].

By contrast, we observed a significant upregulation of two wheat members of the *HMA* transporter family during senescence which have been implicated in Zn and Fe export from the chloroplast to the cytoplasm [12]. The expression of one of these genes, *TaHMA2-like*, was also significantly higher in WT plants than either *gpc-A1* or *gpc-A1/gpc-B2* mutants, which may contribute to the reduced efficiency in Zn and Fe remobilization observed in these mutants. The rice ortholog of this gene, *OsHMA2*, was shown to play an important role in facilitating Zn transport to the rice panicle [54]. These results suggest that nutrient remobilization from the chloroplasts to the cytoplasm for future export to the grain is an early step in wheat monocarpic senescence.

#### Vacuole to cytoplasm transport

Another sink for Zn and Fe during vegetative development is the vacuole, which is utilized as a storage body to prevent plant cell toxicity associated with excess concentrations of these nutrients. In other plant species, VIT and ZIFL transporters have been shown to facilitate nutrient import into the vacuole, while NRAMP transporters have been implicated in Fe export from the vacuole to the cytoplasm. In our study, the expression of both identified wheat *VIT* genes fell during monocarpic senescence, with *TaVIT2* showing a ~4-fold reduction between HD and 22 DAA. However, these differences were not significant for all four statistical tests and were excluded from our DE list. In rice, both OsVIT1 and OsVIT2 proteins localize to the tonoplast and can transport Zn^2+^ and Fe^2+^ across this membrane as part of a vacuolar sequestration strategy [13]. Mutants with reduced or abolished function of both these genes exhibit reduced Zn and Fe concentrations in the leaves coupled with increased levels in the grains, suggesting that a downregulation of these genes during senescence contributes to increased rates of Zn and Fe translocation [13,55].

The *ZIFL* family of transporters is also thought to function in the vacuole. One such transporter in Arabidopsis was implicated in Zn sequestration to the vacuole in vegetative tissues [14], while in barley, a *ZIFL-like* gene is expressed in the aleurone layer of seeds and is induced in the embryo upon foliar Zn application and has been implicated in the regulation of Zn transport to the grain [56]. These potentially distinct roles of the different *ZIFL* genes may explain the differences in transcription profiles observed in this study. *TaZIFL1* was significantly downregulated during senescence, whereas *TaZIFL2-like1* and *TaZIFL7* were significantly upregulated. The rice orthologs of these genes (*OsZIFL2* and *OsZIFL7*) are both upregulated in flag leaves during senescence suggesting that they may promote Zn remobilization during senescence [15]. *OsZIFL1* expression was not detected in rice flag leaves at any stage of development. Further studies will be required to better characterize the function of ZIFL transporters in wheat.

None of the eight *NRAMP* genes identified in wheat were DE between WT and *gpc* mutants and only one (*TaNRAMP2*) was significantly upregulated during senescence (Table 2). This upregulation during senescence is consistent with the known function of this class of transporters, which are thought to facilitate Fe export from the vacuole when remobilization is required during development [57]. However, our finding that most of the wheat *NRAMP* genes were not DE during wheat senescence suggests that these transporters may act in other tissues or stages of developmental stages.

In summary, among the genes involved in the transport for Fe and Zn to and from the vacuole only *TaZIFL7* was DE in both *gpc* mutant genotypes, suggesting a limited effect of the *GPC* genes in the regulation of this group of genes. However, it will still be interesting to investigate the role of *TaZIFL7* and of the other three genes from this group which are DE during senescence.

#### Cytoplasm to phloem transport

For their transport to the grain, nutrients must cross the plasma membrane into the phloem. Members of the *YSL* and *ZIP* families are known to play important roles in the transport of metal-chelate complexes across the plasma membrane in several plant species (Curie et al. 2009) [19]. In wheat, we identified and characterized 14 and 19 members of the YSL (Additional file 1: Figure S8) and ZIP (Additional file 1: Figure S9) families, respectively. Two of the wheat *YSL* genes were upregulated during senescence and one was downregulated, suggesting that they performed different functions (Table 2). However, there is still little evidence from the literature regarding *YSL* function in vegetative Zn and Fe transport, so further experiments will be required to define their role in wheat.

A much clearer link between *GPC1* and Zn and Fe transport during monocarpic senescence was observed for members of the *ZIP* gene family. A total of five *TaZIP* genes were upregulated during senescence and the expression of all five plus *TaZIP5* and *TaZIP10* was significantly higher in WT plants than in either *gpc-A1* or *gpc-A1/gpc-B2* mutant genotypes. *TaIRT-like2* was also DE, but only between WT and the *gpc-A1/gpc-B2* double mutant (Table 2). In some cases, the effect was very large; for example, the expression of both *TaZIP3* and *TaZIP5* were more than 15-fold higher in WT plants than either of the *gpc* mutant genotypes at 22 DAA. These results demonstrate that *GPC* plays a critical role in the regulation of multiple members of this family and that *ZIP* expression is likely to be associated with the remobilization of Zn and Fe from leaves to grain in wheat. A recent study in barley demonstrated that the over-expression of *HvZIP7* resulted in increased Zn accumulation in the grain [21]. The corresponding wheat homologue, *TaZIP7*, was also significantly upregulated by *GPC* during senescence in the current study, suggesting a conserved role for this transporter. For future biotechnological applications, it will be important to determine which of these ZIP transporters represents a limiting step in the remobilization of Zn and Fe from leaves to the grain.

#### PS biosynthesis

It has been hypothesized that a critical limiting factor for nutrient transport to the grain is their solubility in the alkaline environment of the phloem [30]. To prevent precipitation, Fe and Zn ions are transported as a complex with organic ligands or chelators, one critical class of which is the mugineic acid family of PS (Figure 1). We show in this study that several wheat genes encoding key biosynthetic enzymes of this pathway are upregulated during senescence and are differentially regulated by the *GPC* genes. A significant upregulation in *TaNAAT2* expression between HD and 12 DAA and an increase in *TaNAS3* between 12 DAA and 22 DAA suggest an active and possibly coordinated role of these genes during wheat monocarpic senescence. Both *TaNAAT* genes were DE in at least one *gpc* mutant genotype (Table 2) suggesting that they might contribute to the reduced concentrations of Fe and Zn observed in the grains of the *gpc* mutant plants. No significant differences were detected for *TaDMAS1*.

In the rice phloem, Fe and Zn can associate with both NA and DMA, although Zn-NA and Fe-DMA complexes have been shown to be more common [28,29]. *NAS* overexpression has been shown to result in dramatic increases in both Zn and Fe grain content in rice [33,34], making the orthologous genes in wheat interesting targets for biofortification strategies. Multiple transgenic strategies have been successfully applied in rice to increase micronutrient levels in the grain [58], although to date, such strategies have not been described in wheat. The characterization of the different transporter gene families during wheat monocarpic senescence provides the basic information required to select targets for similar biotechnological approaches in this economically important crop species.

In addition to these well-characterized transporter families, we also identified 22 other transporter-related genes which were upregulated between HD and 12 DAA in WT plants, but not in *gpc-A1* mutants (Additional file 1: Table S5). Among these genes, we found one differentially regulated potassium transporter and two sulfate transporters, which are potentially related to the differences identified in K and S concentrations in the grain between genotypes (Additional file 1: Table S1). A similar increase in K and decrease in S, Zn and Fe concentration was also observed in the grains of *GPC-*RNAi transgenic plants with reduced transcriptional levels of all *GPC* genes [5,59]. It will be interesting to investigate whether there is a functional connection between the DE of Fe, Zn, S and K transporters and the effect of the *GPC* genes on their concentration, in the wheat grain.

## Conclusions

In summary, this study confirms that *GPC1* plays a strong role in the regulation of wheat monocarpic senescence, and that it is involved in the upregulation of a large number of genes during the early stages of wheat monocarpic senescence. These genes are likely involved in the plant’s active preparation for the upcoming senescence. Supporting this hypothesis, we found several transporters and genes involved in the biosynthesis of chelators that facilitate Zn and Fe transport through the phloem that were differentially regulated by the *GPC* genes. Among these transporters, members of the large *ZIP* gene family appear to be interesting targets for biotechnological applications and for screening of different natural alleles to improve nutrient remobilization.

## Methods

### Plant material

We previously identified individuals in our tetraploid Kronos TILLING population which carry mutations introducing premature stop codons in *GPC-A1* (W114*, henceforth *gpc-A1*) and *GPC-B2* (W109*, henceforth *gpc-B2*) [51]. Since WT Kronos carries a single nucleotide insertion in the coding region of *GPC-B1* that results in a non-functional protein [44], the *gpc-A1* mutant carries no functional *GPC1* genes.

From the transcriptome dataset, we found that Kronos expresses a *GPC-A2* copy which was not previously reported. We developed *GPC-A2-*specific primers (Forward = 5’–CACCCACCAGCTAGAAGCTC–3’, reverse = 5’–ATCCATGCAATGGTGATGTG–3’) and confirmed the 2AS chromosome arm location using the IWGSC genomic sequence database (IWGSC_CSS_2AS_scaff_5260301). The genomic sequence of *GPC-A2* from Kronos was deposited in GenBank (accession number KM272993). We screened our tetraploid TILLING population for deleterious mutations in *GPC-A2,* but did not find any premature stop codons or splice site mutations. Therefore, the *gpc-B2* mutation alters the dosage of *GPC2* transcripts but does not result in a complete loss-of-function for *GPC2*.

The M_3_ individuals carrying these TILLING mutations were backcrossed twice to WT Kronos to reduce the background mutational load and were combined to create BC_2_F_2_ sibling *gpc-A1* and *gpc-B2* single mutants, as well as *gpc-A1/gpc-B2* double mutant plants and WT siblings carrying no TILLING mutations. Plants were grown in the greenhouse under long day conditions and single, entire flag leaves were harvested from four biological replicates of WT, *gpc-A1* and *gpc-A1/gpc-B2* plants at HD, 12 DAA and 22 DAA and immediately frozen in liquid nitrogen for RNA-seq library construction. BC_2_F_3_ seeds were harvested for field trials. Seeds from all three mutant genotypes (*gpc-A1* PI 673414, *gpc-B2* PI 673413 and *gpc-A1/gpc-B2* PI 673415) were submitted to the Germplasm Resources Information Network (http://www.ars-grin.gov/npgs/).

### Field experiments

During the 2011–2012 growing season, field experiments to determine the phenotype of the *GPC* mutants were carried out in one location in the USA (Davis, CA, UCD-2012) and in two locations in Israel (Newe Ya'ar Regional Research Center, NY-2012 and Tel-Aviv University, TAU-2012). During the 2012–13 growing season one experiment was carried out at Newe Ya'ar Regional Research Center, NY-2013. All experimental plots were arranged in a randomized complete block design. Full details of planting date, soil components, precipitation, irrigation and fertilization are provided for each field trial in Additional file 1: Table S6.

Five traits were evaluated in each field experiment: 1) *Dry spike weight*: Plants were tagged at anthesis and spikes were collected at 35, 42 and 49 DAA. There were no significant differences in anthesis date among genotypes in any of the field experiments. Spikes were then dried for 48 h at 70°C and the average weight of 10 spikes was determined. 2) *Relative chlorophyll content*: This parameter was determined in flag leaves using a hand-held chlorophyll meter (SPAD-502, Minolta, Milton Keynes, UK) for experiments performed in the USA and a CCM-200 (Opti-Science, Hudson, NH) for experiments performed in Israel. All CCM-200 results were normalized to SPAD values [60]. Each value corresponds to the average of ten readings across the flag leaf, presented as relative SPAD units. 3) *Kernel weight*: Completely senesced plants were individually harvested and each spike was threshed separately. Grains from the tagged main spike were dried to a constant weight at 70°C and dry weights were obtained and reported as thousand kernel weight ([total grain weight/ number of grains]*1000). 4) *Grain protein content*: this parameter was measured from mature grain using a grain analyzer-Perten IM9200 (Perten Instruments AB, H.Q. Stockholm, Sweden) at the University of California, Davis. Ten biological replicates were used in the UCD-2012 experiment and four each for NY-2012 and TAU-2012 experiments. 5) *Micronutrient determinations*: The levels of 11 different micronutrients, including Zn and Fe were measured in the mature, harvested grain using ICP-MS (Inductively Coupled Plasma - Mass Spectrometry) (Agilent Technologies, Santa Clara, CA, USA) at the University of California, Davis. Five biological replicates of each genotype from the UCD-2012 and TAU-2012 experiments were analyzed.

### RNA-seq library construction and sequencing

Flag leaves from four biological replicates of three different genotypes (WT, *gpc-A1,* and *gpc-A1/gpc-B2*, BC_2_F_2_ generation) were collected at three different time points (HD, 12 DAA and 22 DAA). Samples were ground to a fine powder using a pestle and mortar in liquid nitrogen and RNA samples were extracted using the Spectrum™ Plant Total RNA Kit (Sigma-Aldrich, St. Louis, MO). Concentration and purity of total RNA was confirmed on a NanoDrop® ND-1000 spectrophotometer and RNA integrity was evaluated by standard agarose gel electrophoresis. RNA-seq libraries were constructed using the TruSeq™ RNA sample preparation kit (Illumina, San Diego, CA) and their quality determined by running samples on a high-sensitivity DNA chip on a 2100 Bioanalyzer (Agilent Technologies, Santa Clara, CA). For each of the four biological replicates, the nine genotype/time point samples were tagged with a unique index to allow multiplexing and were sequenced in three lanes on an Illumina HiSeq2000 sequencer, using the 50 bp single read module at the UC Davis Genome Center (total 12 Illumina lanes for the 36 samples). On average we obtained 36.6 M raw reads per sample (Additional file 1: Table S2).

### Bioinformatics analysis

#### Aligning sample reads to the reference genome

As a reference for read mapping we used the current draft of sequenced genomic contigs from flow-sorted chromosome arms of the hexaploid wheat variety “Chinese Spring” generated by the IWGSC [48] and hosted by Unité de Recherche Génomique (URGI, http://wheat-urgi.versailles.inra.fr/). Since our experiments are in tetraploid wheat, only the sequences from the A and B genomes were used as a reference. We aligned reads from each sample to this reference using *GSNAP(l)*, a splicing-aware aligner (version 05-09-2013, default parameters except -m 2 -n 1 -N 1 -A sam [61]) to generate SAM mapping files for each sample.

#### Creating a putative transcribed region annotation track

At the time of our analysis, the wheat genomic reference lacked genic annotation required to identify expressed regions within our mapping results. Therefore, we created a set of putative transcribed ranges using a separate comprehensive set of wheat transcript data. We compiled a total of 286,814 wheat transcripts, which were derived from non-redundant sequences from four wheat transcriptome assemblies combined with additional wheat sequences taken from several other public sequence databases (described in Krasileva et al. [49] and in http://maswheat.ucdavis.edu/Transcriptome/index.htm). Using *GMAP* (Version 05-09-2013, default parameters except -n 1 --nofails --cross-species -f samse -x 0 [62], we mapped this set of transcripts separately, to the A-homoeologous group chromosomes contigs, and then to the B-homoeologous group chromosome contigs. This separate mapping approach was implemented to ensure that the aligner found all homoeologous genes in each dataset. *Bedtools cluster* (−d 0) was then used to merge overlapping aligned regions, followed by *bedtools merge* to merge overlapping regions into a single putative transcribed region. The resulting GFF file consisted of 135,571 genomic ranges, each representing the genomic contig identifier and the start and end coordinate of the putative transcribed region. These genomic ranges will be referred to hereafter as loci. Despite the inclusion of all current publicly available wheat transcripts, there remained a small proportion of 50 bp Illumina reads which mapped outside of the genomic regions defined in our GFF file, which likely represent transcribed mRNA expressed in our biological samples, but absent from our transcriptome dataset. To expand our genomic loci to include these regions, we mapped the reads from one biological replicate of WT samples at each time point (HD, 12 DAA and 22 DAA) using *GSNAP* as described above. Using these alignments, we defined novel transcribed genomic regions as those with a read depth ≥10. These aligned regions were then clustered in cases where they were separated by ≤1 kbp using *bedtools cluster* (−d 1000) and overlapping regions were merged using *bedtools merge* to create a single putative transcribed region. This resulted in the identification of 4,257 additional loci, which were added to the existing GFF file, giving a total of 139,828 genomic loci. Loci identifiers are from URGI (http://wheat-urgi.versailles.inra.fr/) and are listed in Additional file 2 along with the corresponding Ensembl locus ID and associated high-confidence protein coding gene, where available (http://plants.ensembl.org/Triticum_aestivum/Info/Index).

#### Counting sample reads overlapping putative transcribed regions

Raw count values were determined using *HTSeq count* [63] (−m union) using the generated GFF file and individual SAM alignment files for each sample. We considered only those reads mapped uniquely within the regions defined within the GFF file for differential expression analysis. Before normalization, we used a custom R package (*noleaven*) to remove genomic loci which had zero or extremely low coverage across all genotype/time point samples to test for DE. This reduces the number of statistical tests, and therefore the required corrections for multiple-testing. Further details and code are available online (http://topherconley.github.io/noleaven/). In this analysis, only loci which had more than three reads mapping from at least two biological replicates of any genotype/time point sample were retained. This resulted in the removal of 59,960 loci, leaving 80,168 loci with counts above this threshold for further consideration.

Since the total number of reads varied between biological samples, raw counts were normalized using the R/Bioconductor software package *DESeq* (Version 1.12.1 [64], R Version 2.14.2). After normalization, we performed 9 different pairwise comparisons: within the WT genotype we compared HD *vs.* 12 DAA, HD *vs.* 22 DAA, and 12 DAA *vs.* 22 DAA; and within each time point we compared WT *vs. gpc-A1* and WT *vs. gpc-A1/gpc-B2* mutants. We applied four statistical tests and we only considered a locus differentially expressed if it was significant for all four tests simultaneously using the thresholds described below. First, pairwise comparisons using *DESeq* and *edgeR* [65] were made between samples. The *P*-values generated by both analyses were adjusted for false discovery rates (FDR), using the procedure of Benjamini and Hochbergh [66] as implemented in the R/Stats package, using a cutoff of adjusted *P* ≤ 0.01 for significance. Throughout the paper, both *DESeq* and *edgeR* results refer to the FDR-adjusted *P* values. We also applied a Mann–Whitney-Wilcoxon (MWW) test (*P* ≤ 0.05) and a *t*-test (*P* ≤ 0.01). The requirement of significance in all four tests is a conservative approach that has the effect of reducing the false positive rate at the expense of a reduction in the power to detect genuinely DE transcripts (false negatives). To give the readers the opportunity to reanalyze this data using less stringent approaches we present all count data with the *P* values for all four tests for the nine pairwise comparisons (Additional file 2).

### Functional annotation of genomic loci

To determine the function of genes within our differentially regulated gene sets, we isolated the longest contig from each of the 80,168 genomic loci included in our reference set, including the predicted ORF as described in Krasileva et al. [49]. We performed a BLASTX against the nr protein database (NCBI 2013–10 release) and also screened the translated ORF for each contig where this was available against the Pfam database version 27.0 with InterProScan version 4.8 to discern the existence of conserved protein domains. The resulting information was used to infer GO terms associated with each genomic locus using BLAST2GO version 2.6.5. We obtained an annotated function for 41,474 (51.7%) genomic loci and used the ‘R’ package *TopGO* version 2.14.0 to perform an enrichment analysis among the differentially regulated gene sets. “Biological Process” terms were obtained and significance values for enrichment were calculated using ‘classic’ Fishers’ exact test, implemented in *TopGO*.

### qRT-PCR validation

We synthesized cDNA using the Quantitect reverse transcription kit (Qiagen, Valencia, CA), using RNA extracted for RNA-seq library construction as a template. qRT-PCR reactions were performed as described previously [39] and using the primers described in Additional file 1: Table S7.

### Phylogenetic analysis

Proteins from putative transporters within each family were aligned using MUSCLE and a neighbor-joining tree constructed using pairwise deletions and 1,000 bootstrap iterations with the program MEGA 5.0. To simplify the trees, only one homoeologue of each wheat gene was included.

### Availability of supporting data

The raw data sets including sequencing reads were deposited in the National Center for Biotechnology Information’s Gene Expression Omnibus (accession number GSE60635 http://www.ncbi.nlm.nih.gov/geo/query/acc.cgi?acc=GSE60635).

## Additional files

Additional file 1: Figure S1. Relative chlorophyll content of flag leaves taken from (a) TAU-2012, (b) NY-2012 and (c) NY-2013 experiments. **Figure S2.** Spike and grain phenotypes in tetraploid *GPC* mutants. **Figure S3.** Phenotype of WT, *gpc-A1* and *gpc-A1/gpc-B2* TILLING mutants at 22 DAA (a-c) and at 60 DAA (d-f). **Figure S4.** Principal component analyses of biological samples according to normalized expression values per locus of all genes by time point (a-c) and genotype (d-f). **Figure S5.** Relative expression of *GPC* genes in WT, *gpc-A1* and *gpc-A1/gpc-B2* lines. **Figure S6.** HMA phylogeny. **Figure S7.** ZIFL phylogeny. **Figure S8.** YSL phylogeny. **Figure S9.** ZIP phylogeny. **Figure S10.** Validation of six transporter genes by qRT-PCR analysis. **Table S1.** Micronutrient concentrations in UCD-2012 and TAU-2012 experiments. **Table S2.** Summary of raw and trimmed Illumina sequencing reads and mapping rates from each biological replicate. **Table S3.** 26 genes commonly differentially regulated during senescence between the current study and Gregersen & Holm (2007) [40]. **Table S4.** 196 affymetrix contigs from Jukanti et al. 2008 [38] also differentially expressed during senescence. **Table S5.** List of 33 loci significantly upregulated between HD and 12 DAA in WT plants but not in *gpc-A1* plants. **Table S6.** Details of field experiments. **Table S7.** qRT-PCR primers used in this study and their efficiency. Additional file 2:
**RNA-seq data.** Table of genomic contigs, associated Ensembl contig and protein-coding gene, NCBI BLASTX result, normalized count values, trend during senescence and whether the gene is included in the *GPC-A1* or *GPC-A1/GPC-B2* regulated set. For each pairwise comparison, fold-change and statistical results from each test is described.

## Abbreviations

GPC: Grain Protein Content
FRO: Ferric chelate reductase
IRT: Iron Regulated Transporter
ZIP: ZRT, IRT-like Protein
YSL: Yellow Stripe-Like
PS: Phytosiderophore
HMA: Heavy Metal ATPase
ZIFL: Zinc Induced Facilitator Like
NA: Nicotianamine
NAS: Nicotianamine Synthase
DMA: 2’-deoxymugineic acid
NAAT: Nicotianamine Aminotransferase
DMAS: DMA synthase
WT: Wild type
UCD: UC Davis
TAU: Tel Aviv University
NY: Newe Ya’ar
HD: Heading Date
DAA: Days after anthesis
IWGSC: International Wheat Genome Sequencing Consortium
PCA: Principal component analysis
DE: Differential expression
GO: Gene ontology
FDR: False discovery rate
MWW: Mann–Whitney-Wilcoxon
