# Supplementary material for: Regulation of Zn and Fe transporters by the GPC1 gene during early wheat monocarpic senescence
Source: BMC Plant Biol. 2014 Dec 19;14:368. doi: 10.1186/s12870-014-0368-2 (PMC4302714; doi:10.1186/s12870-014-0368-2)
Supplement: Additional file 1: Figure S1. — Relative chlorophyll content of flag leaves taken from (a) TAU-2012, (b) NY-2012 and (c) NY-2013 experiments. Figure S2. Spike and grain phenotypes in tetraploid GPC mutants. Figure S3. Phenotype of WT, gpc-A1 and gpc-A1/gpc-B2 TILLING mutants at 22 DAA (a-c) and at 60 DAA (d-f). Figure S4. Principal component analyses of biological samples according to normalized expression values per locus of all genes by time point (a-c) and genotype (d-f). Figure S5. Relative expression of GPC genes in WT, gpc-A1 and gpc-A1/gpc-B2 lines. Figure S6. HMA phylogeny. Figure S7. ZIFL phylogeny. Figure S8. YSL phylogeny. Figure S9. ZIP phylogeny. Figure S10. Validation of six transporter genes by qRT-PCR analysis. Table S1. Micronutrient concentrations in UCD-2012 and TAU-2012 experiments. Table S2. Summary of raw and trimmed Illumina sequencing reads and mapping rates from each biological replicate. Table S3. 26 genes commonly differentially regulated during senescence between the current study and Gregersen & Holm (2007) [40]. Table S4. 196 affymetrix contigs from Jukanti et al. 2008 [38] also differentially expressed during senescence. Table S5. List of 33 loci significantly upregulated between HD and 12 DAA in WT plants but not in gpc-A1 plants. Table S6. Details of field experiments. Table S7. qRT-PCR primers used in this study and their efficiency. [file 12870_2014_368_MOESM1_ESM.pdf]

Additional file 1

Regulation of Zn and Fe transporters by the *GPC1* gene during early wheat monocarpic senescence

Stephen Pearce, Facundo Tabbita, Dario Cantu, Vince Buffalo, Raz Avni, Hans Vazquez-Gross, Rongrong Zhao, Christopher J. Conley, Assaf Distelfeld and Jorge Dubcovsky.

This PDF file includes:

Figures S1 to S10.
Tables S1 to S7.

Figure S1. Relative chlorophyll content of flag leaves taken from (a) TAU-2012, (b) NY-2012 and (c) NY-2013 experiments.

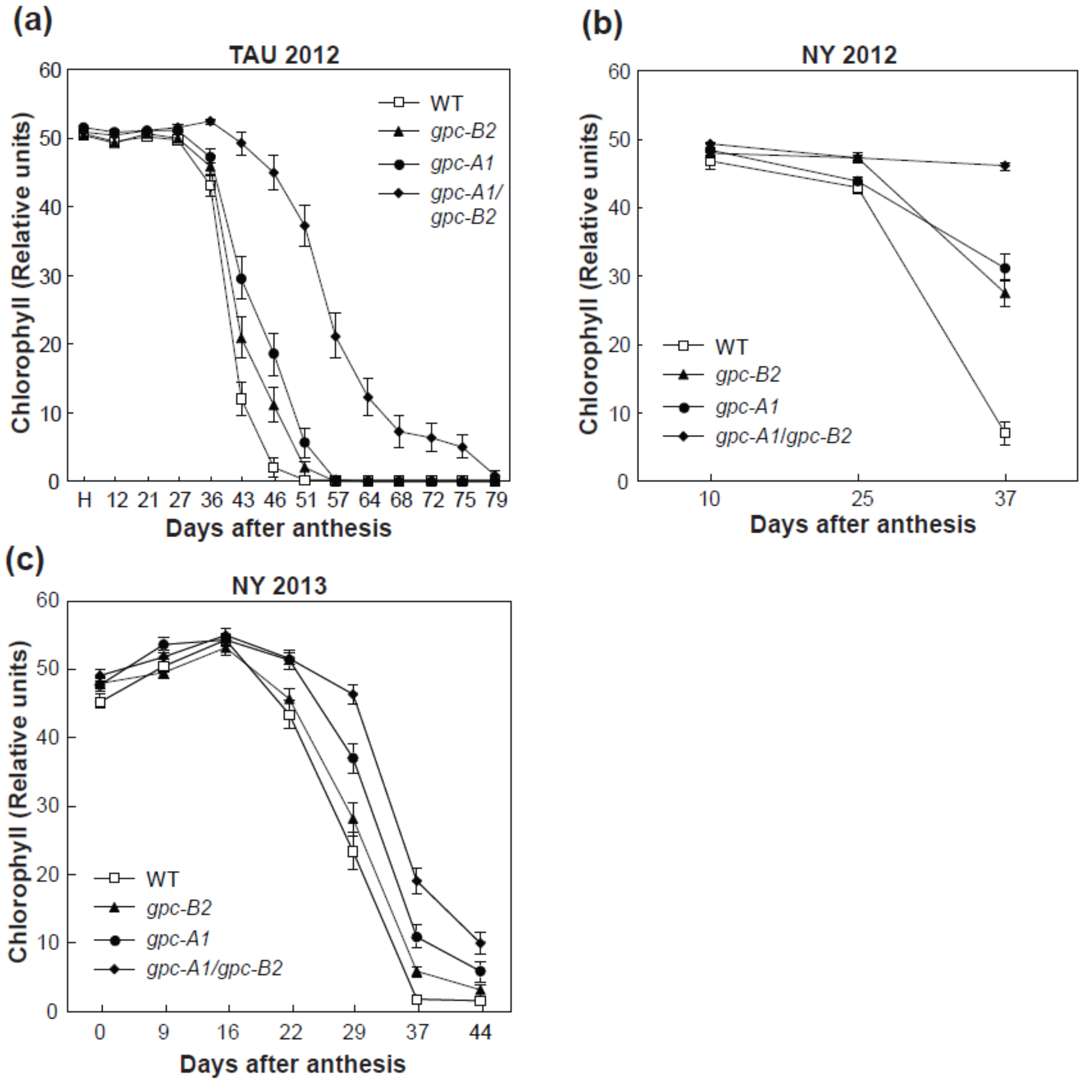

Figure S2. Spike and grain phenotypes in tetraploid *GPC* mutants. (a) Thousand kernel weight from three field experiments. Two-way factorial ANOVA was performed for TKW including location and genotype (*P*=0.02) (b) Dry spike weight at three timepoints from the UCD 2012 experiment. *** = *P*<0.001, difference when compared to WT control sample from Dunnett's test. UCD = UC Davis 2012 experiment, TAU = Tel Aviv University 2012 experiment, NY = Newe Ya'ar research center 2013 experiment. DAA = Days after anthesis.

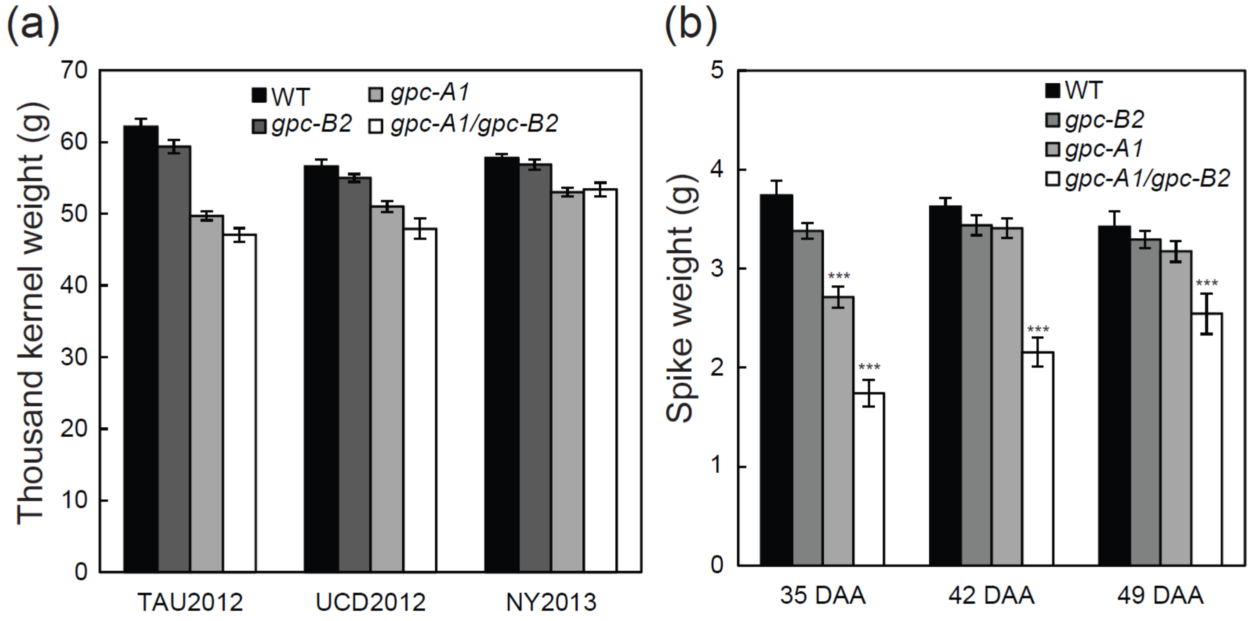

Figure S3. Phenotype of WT, *gpc-A1* and *gpc-A1/gpc-B2* TILLING mutants at 22 DAA (a-c) and at 60 DAA (d-f). In all panels, order of plants from left to right is WT, *gpc-A1*, *gpc-A1/gpc-B2*.

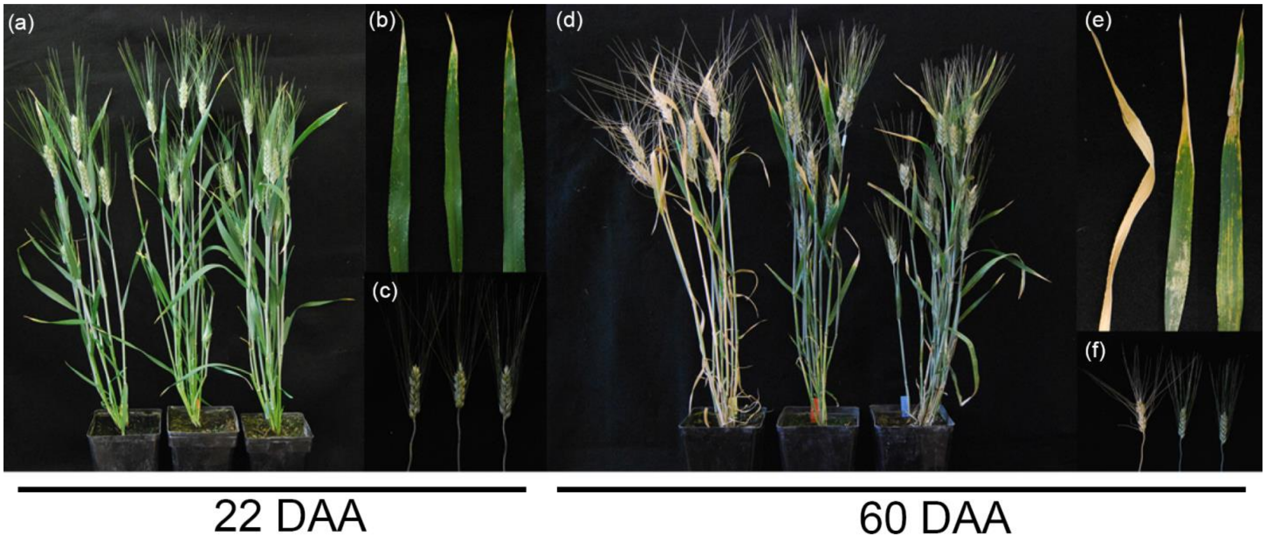

Figure S4. Principal component analyses of biological samples according to normalized expression values per locus of all genes by time point (a-c) and genotype (d-f).

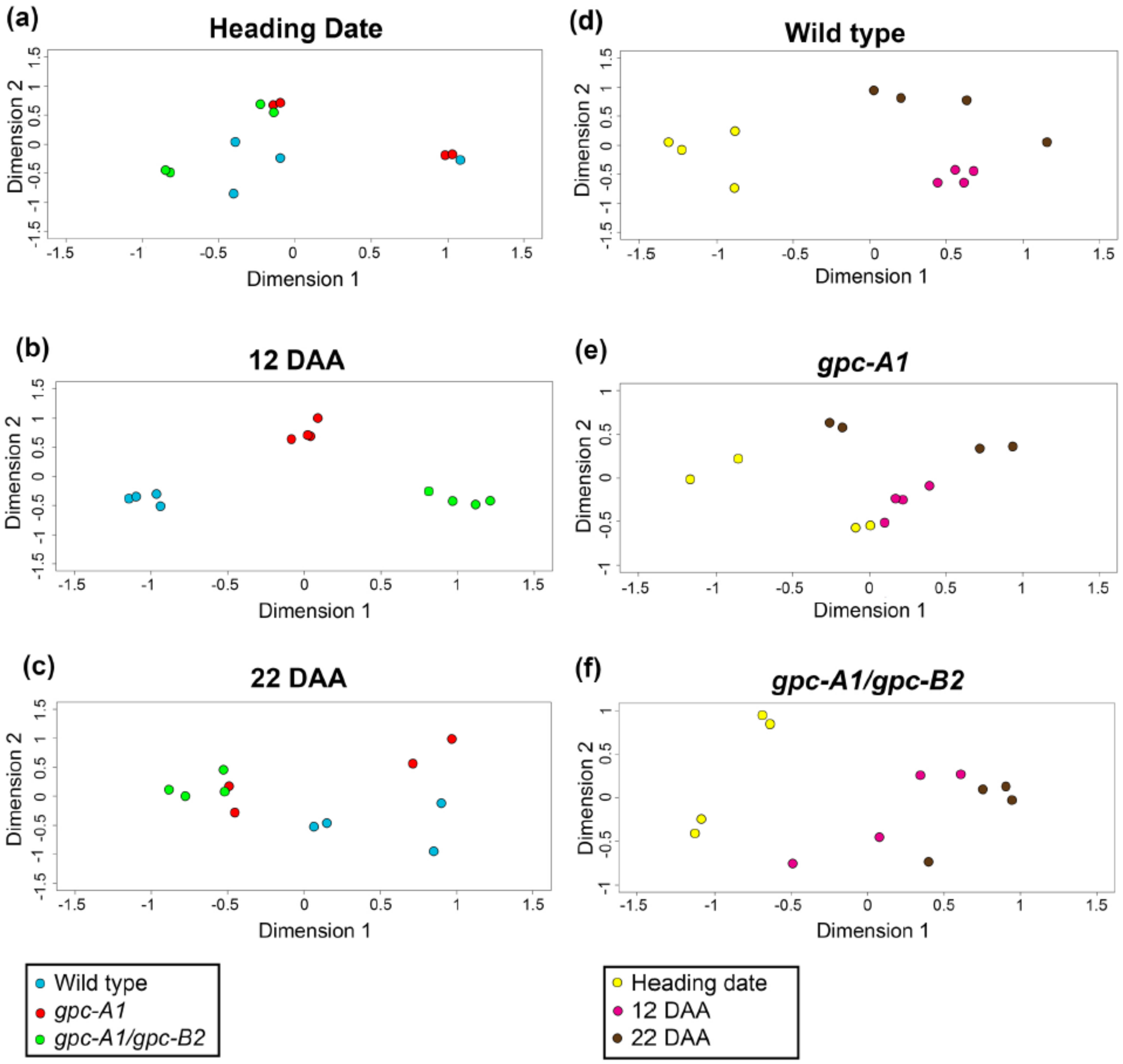

Figure S5. Relative expression of *GPC* genes in WT, *gpc-A1* and *gpc-A1/gpc-B2* lines. Expression presented as RPKM from four biological replicates ±SE of normalized reads mapping to the loci representing each *GPC* gene.

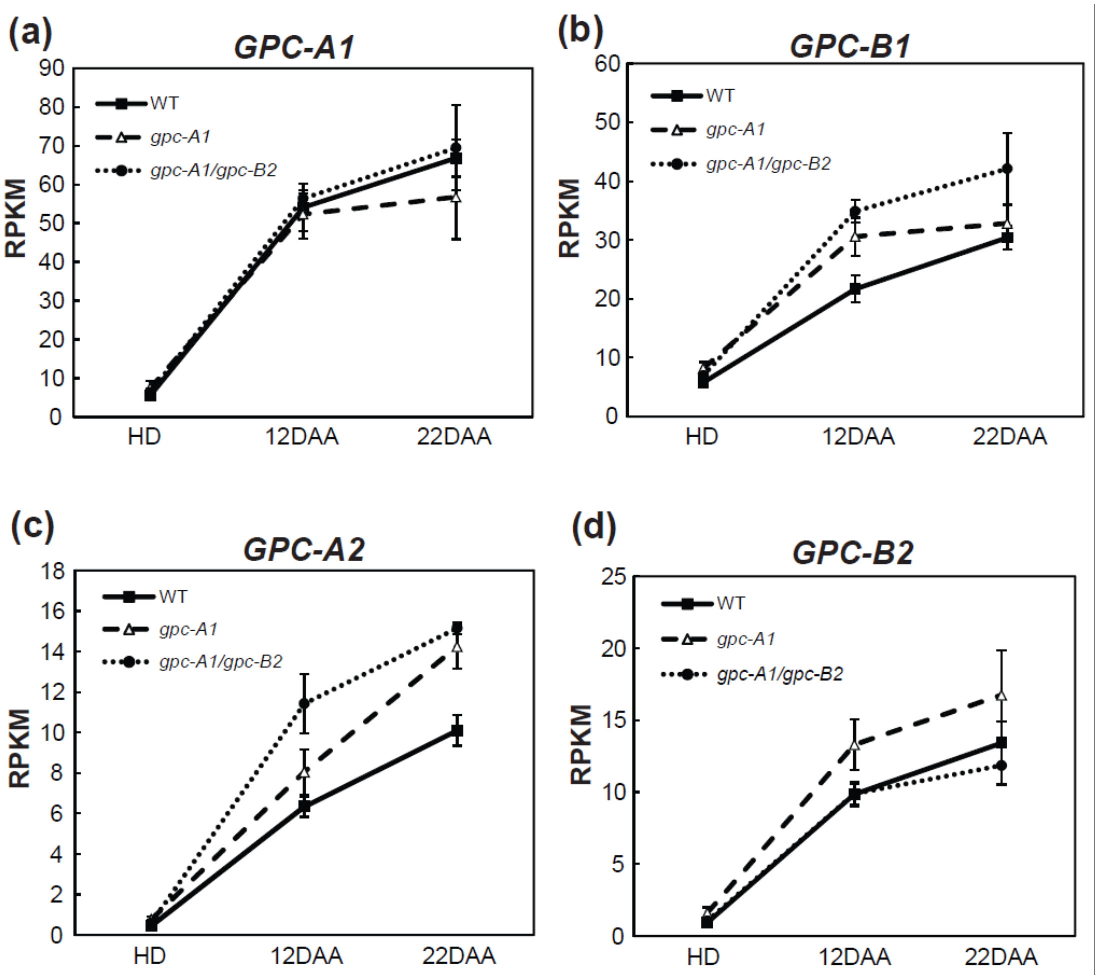

Figure S6. HMA phylogeny. Rice protein IDs (blue): OsHMA1 (NP_001058417.1), OsHMA2 (BAM36049.1), OsHMA3 (BAJ25742.1). Wheat protein IDs (red): TaHMA1 (Traes_7BL_041308E74), TaHMA1-like (Traes_5AL_C89EEBE50), TaHMA2 (Traes_7AL_8304348B7), TaHMA2-like (Traes_7AL_6AE850114), TaHMA3 (Traes_5BL_D6C3DC326). Arrows indicate up or downregulation during senescence in WT plants. * denotes differential expression in at least one *GPC* mutant genotype.

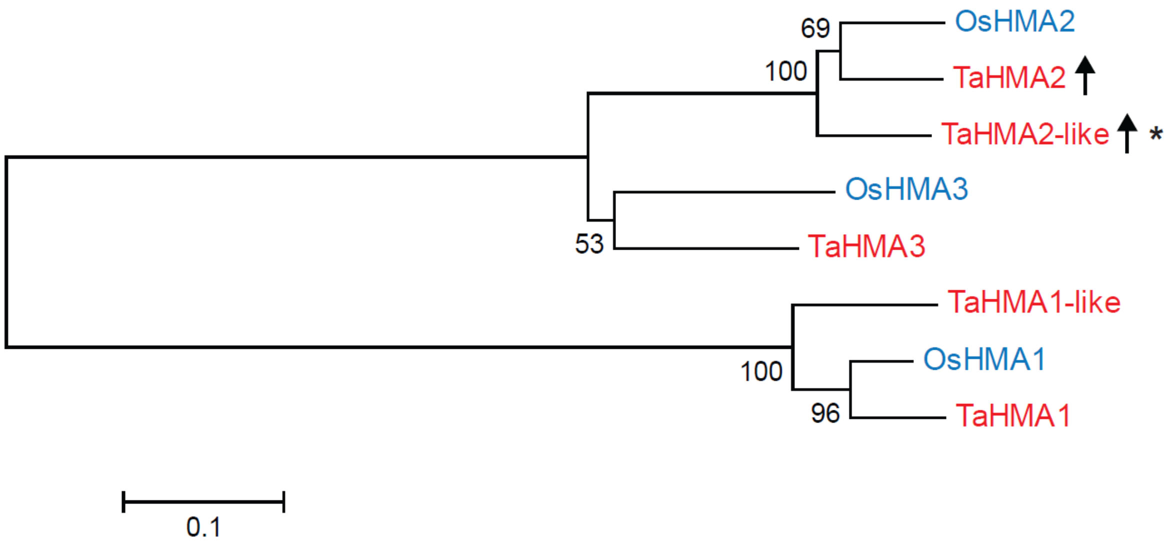

Figure S7. ZIFL phylogeny. Rice protein IDs (blue): OsZIFL1 (NP_001042690.1), OsZIFL2 (NP_001042748.1, OsZIFL3 (BAG89474), OsZIFL4 (NP_001065681.1), OsZIFL5 (NP_001065682.1), OsZIFL6 (ABA91327.1), OsZIFL7 (ABG22349.1), OsZIFL8 (ABA91341.1), OsZIFL9 (ABA95719), OsZIFL10 (NP_001066084.1), OsZIFL11 (NP_001066085.1), OsZIFL12 (ABG21873.1), OsZIFL13 (ABA95731.1). Wheat protein IDs (red): TaZIFL1 (Traes_3AS_C25151458), TaZIFL1-like (Traes_3AS_02DE247DA), TaZIFL2 (Traes_5BL_A0B9DE62E), TaZIFL3 (Traes_4BS_1DCF82CB7), TaZIFL7 (Traes_5AL_37BFFFD9E), TaZIFL8 (Traes_4AL_5C7A4DA54), TaZIFL9 (Traes_5AL_0599F7BC5). TaZIFL2-like1 was not included in the phylogeny. Arrows indicate up or downregulation during senescence in WT plants. * denotes differential expression in at least one *GPC* mutant genotype.

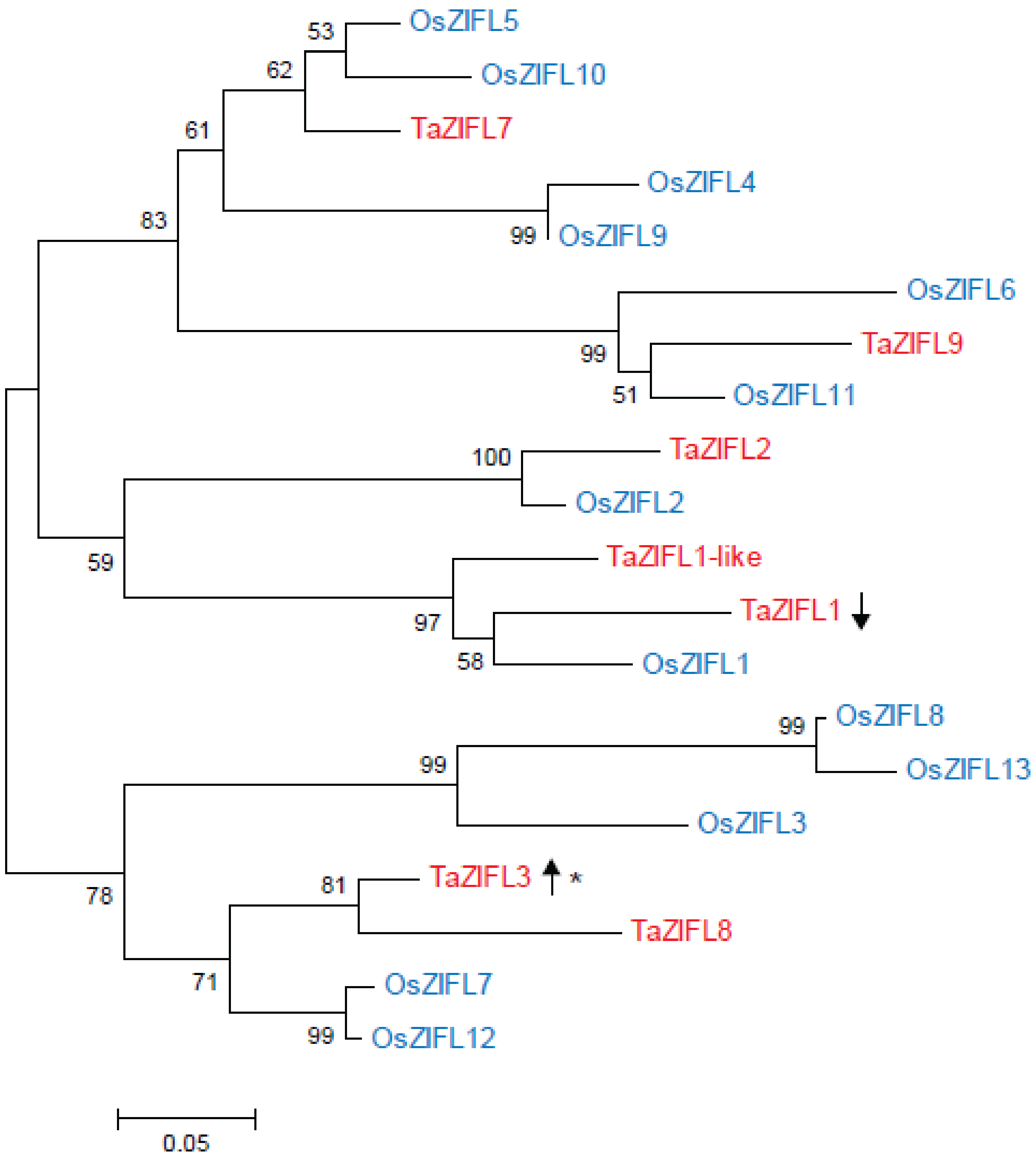

Figure S8. YSL phylogeny. Rice protein IDs (blue): OsYSL1 (NP_001042539.1), OsYSL2 (BAD90812.1), OsYSL3 (BAE91883.1), OsYSL4 (BAE91884.1), OsYSL5 (BAE91885.1), OsYSL6 (BAE91886.1), OsYSL7 (BAE91887.1), OsYSL8 (BAE91888.1), OsYSL9 (BAE91889.1), OsYSL10 (BAE91890.1), OsYSL11 (BAE91891.1), OsYSL12 (BAE91892.1), OsYSL13 (BAE44204.1), OsYSL14 (BAE44205.1), OsYSL15 (BAE91893.1), OsYSL16 (BAE91894.1), OsYSL17 (BAE91895.1), OsYSL18 (BAE91896.1). Wheat protein IDs (red): TaYSL1 (Traes_3B_17BC3E1E2), TaYSL2 (Traes_6BL_3DD0BA741), TaYSL6 (Traes_2BL_0CBCC13AD), TaYSL9 (Traes_2AL_CFCA01C76), TaYSL10 (Traes_6AL_5642D5B44), TaYSL11 (Traes_2BL_68E0CA743), TaYSL12 (Traes_2BL_2BE05F104), TaYSL13 (Traes_2AL_F707FF2C3), TaYSL13-like (Traes_2BL_A14EA5AE4), TaYSL14 (Traes_6AL_7FB45D4DE), TaYSL15 (Traes_6AL_E36FCEF64), TaYSL15-like (Traes_1BL_CA93E6359), TaYSL16 (Traes_2BL_4A1181B731), TaYSL18 (Traes_2BL_6C5206B6D). Arrows indicate up or downregulation during senescence in WT plants. * denotes differential expression in at least one *GPC* mutant genotype.

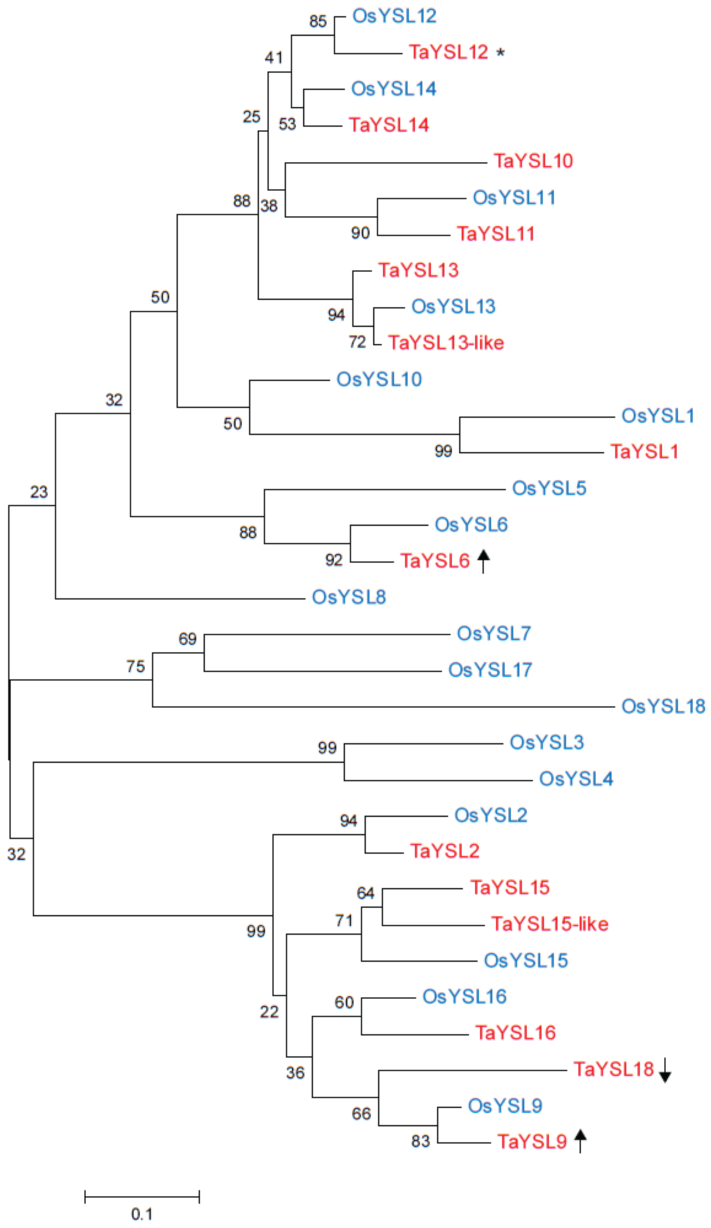

Fig. S9. ZIP phylogeny. Rice protein IDs (Blue): OsIRT1 (NP_001050853.1), OsIRT2 (ABF98082.1), OsZIP1 (NP_001045540.1), OsZIP2 (NP_001050351.1), OsZIP3 (NP_001053851.1), OsZIP4 (NP_001061245.1), OsZIP5 (NP_001055818.1), OsZIP6 (NP_001054742.1), OsZIP7 (NP_001054875.1), OsZIP8 (A3BI11.1), OsZIP9 (Q0DHE3.3), OsZIP10 (EEC80824.1), OsZIP11 (NP_001055173.1), OsZIP13 (NP_001046190.1), OsZIP14 (NP_001062003.1), OsZIP16 (XP_006659010.1). Wheat protein IDs (red): TaIRT-like1 (Traes_4AL_9D79BE8FB), TaIRT-like2 (Traes_4BS_6527BBD54), TaZIP10 (Traes_7AL_A13A246B4), TaZIP10-like1 (Traes_7AL_F1D611563), TaZIP13-like1 (Traes_7BL_12C63350C), TaZIP13-like2 (Traes_2BS_9D8F265EC), TaZIP13-like3 (Traes_2AL_BE05B34FF), TaZIP1 (ABF55691.1), TaZIP2 (CAJ19368.1), TaZIP2-like (Traes_6AS_75C6428051), TaZIP3 (AAW68439.1), TaZIP5 (Traes_4AS_F5F7D2A8D), TaZIP6 (AK333945.1), TaZIP7 (ABF55692.1), TaZIP11 (Traes_1AS_7DC2CB902), TaZIP14 (AK331623.1), TaZIP15 (Traes_6AS_4E1D574BC), TaZIP16 (Traes_7AL_DFE86911E). Arrows indicate up or downregulation during senescence in WT plants. * denotes differential expression in at least one *GPC* mutant genotype.

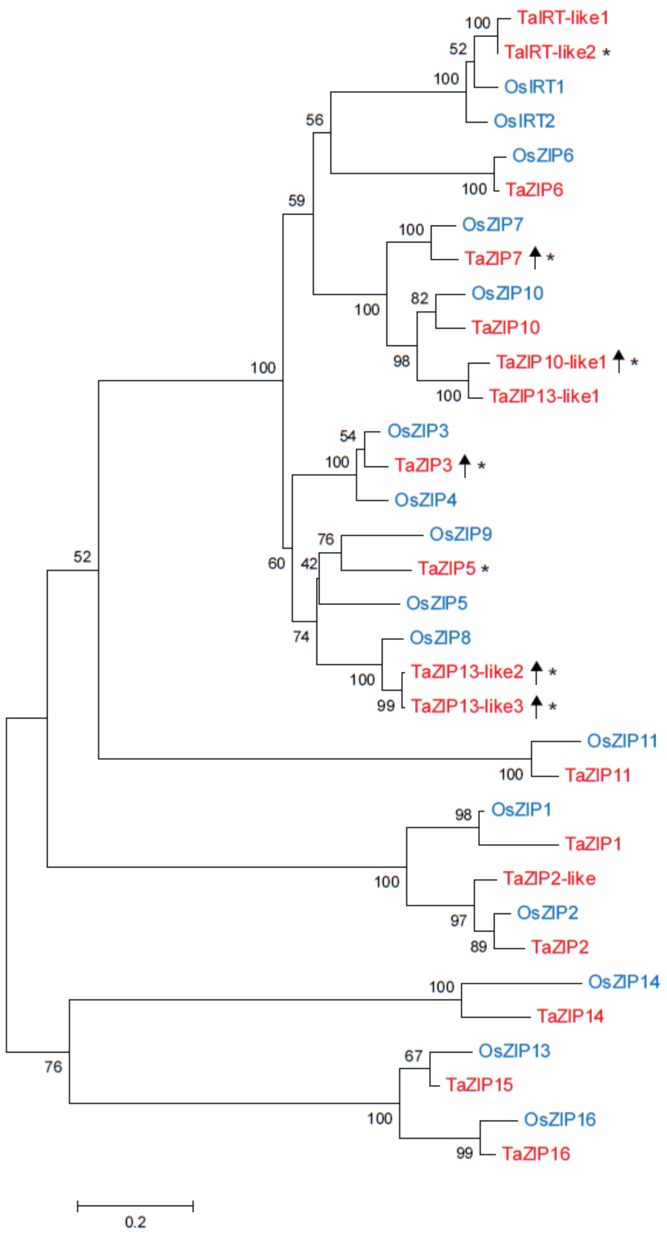

Figure S10. Validation of six transporter genes by qRT-PCR analysis. Side-by-side comparisons of the relative expression levels of each gene as assayed using RNA-seq (Normalized counts +/- SE) and qRT-PCR (fold-*ACTIN* +/- SE).

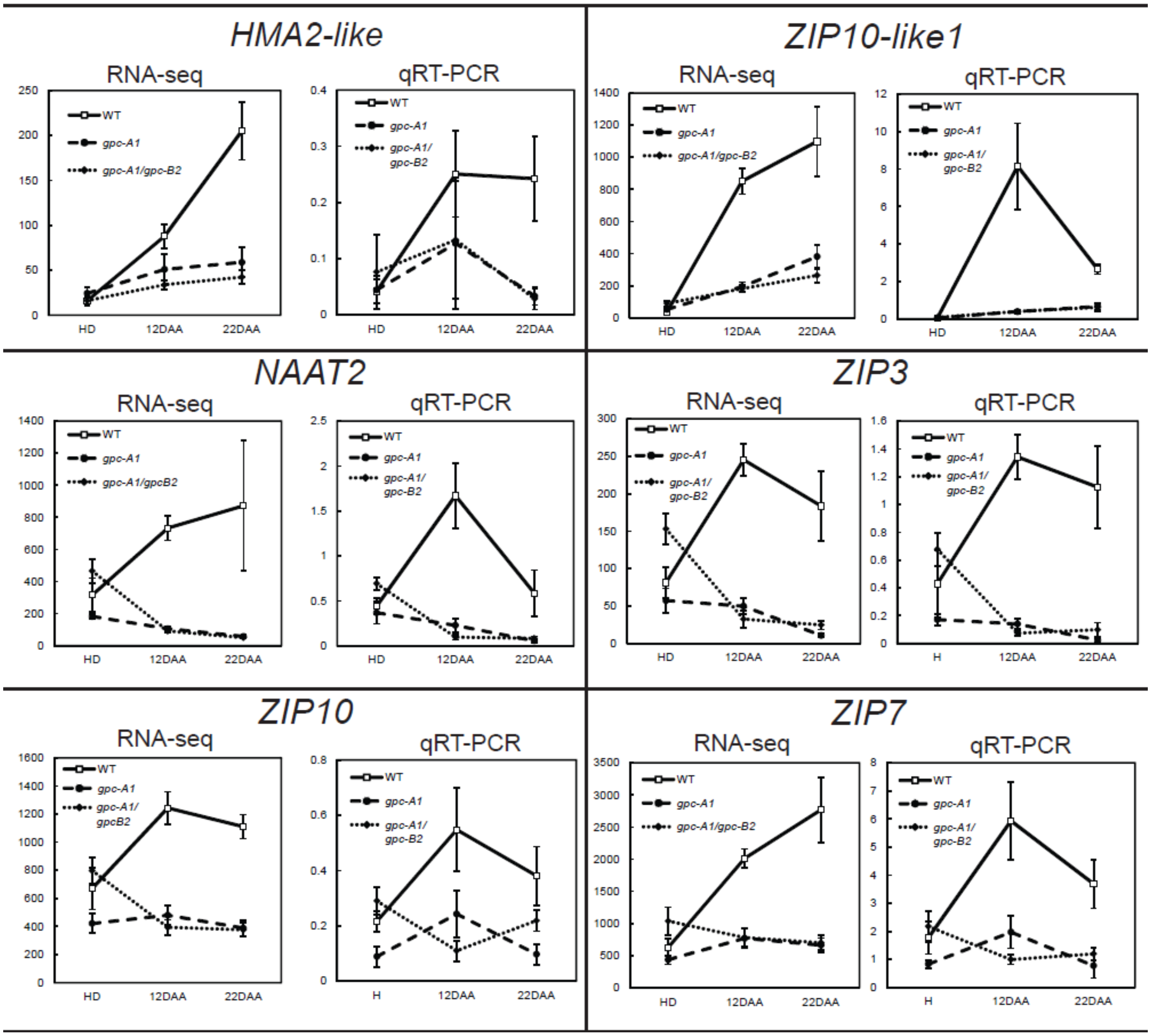

Table S1. Micronutrient concentrations in UCD-2012 and TAU-2012 experiments. GPC and micronutrient grain content for WT, single mutants and double mutant *GPC* knockout plants. Different letters denote significant differences between genotypes determined by Tukey-HSD test (P <0.05).

		WT	*gpc-B2*	*gpc-A1*	*gpc-A1/gpc-B2*
**N (g/kg)**	UCD	29.78 ± 0.3^{a}	29.6 ± 0.3^{a}	23.16 ± 0.2^{b}	26.17 ± 0.7^{c}
	TAU	27.76 ± 0.4^{a}	29.49 ± 0.5^{a}	23.28 ± 0.6^{b}	24.4 ± 0.4^{b}
**P (g/kg)**	UCD	3.7 ± 0.0^{a}	3.58 ± 0.1^{a}	3.62 ± 0.0^{a}	4.15 ± 0.1^{b}
	TAU	4.13 ± 0.1^{a}	4.22 ± 0.1^{a}	4.2 ± 0.0^{a}	4.3 ± 0.1^{a}
**K (g/kg)**	UCD	4.88 ± 0.1^{a}	5.05 ± 0.1^{a}	5.78 ± 0.1^{b}	6.5 ± 0.9^{c}
	TAU	4.79 ± 0.1^{a}	4.53 ± 0.1^{a}	6.38 ± 0.1^{b}	6.15 ± 0.2^{b}
**S (ppm)**	UCD	1848.00 ± 13.2^{a}	1774.00 ± 21.6^{b}	1564.00 ± 11.7^{c}	1737.00 ± 24.1^{b}
	TAU	1783.75 ± 22.5^{b}	1866.25 ± 17.5^{a}	1690.00 ± 26.8^{c}	1777.5 ± 11.1^{b}
**B (ppm)**	UCD	2.42 ± 0.2^{a}	2.75 ± 0.1^{b}	2.94 ± 0.2^{b}	3.36 ± 0.1^{c}
	TAU	0.93 ± 0.0^{a}	1.3 ± 0.2^{ab}	1.4 ± 0.2^{b}	1.35 ± 0.1^{ab}
**Ca (g/kg)**	UCD	0.26 ± 0.1^{ab}	0.25 ± 0.0^{a}	0.28 ± 0.1^{b}	0.36 ± 0.1^{c}
	TAU	0.41 ± 0.0^{a}	0.42 ± 0.1^{a}	0.45 ± 0.0^{a}	0.57 ± 0.1^{b}
**Mg (g/kg)**	UCD	1.41 ± 0.1^{a}	1.44 ± 0.0^{ab}	1.51 ± 0.1^{b}	1.67 ± 0.0^{c}
	TAU	1.33 ± 0.1^{a}	1.41 ± 0.0^{b}	1.35 ± 0.0^{ab}	1.39 ± 0.0^{ab}
**Zn (ppm)**	UCD	50.3 ± 1.1^{a}	48.09 ± 2.1^{a}	39.42 ± 0.6^{b}	42.63 ± 1.2^{b}
	TAU	53.56 ± 4.7^{b}	61.54 ± 5.2^{a}	45.28 ± 2.8^{c}	40.93 ± 2.9^{c}
**Mn (ppm)**	UCD	34.68 ± 1.8^{bc}	32.71 ± 1.1^{ab}	28.52 ± 0.7^{a}	37.85 ± 2.0^{c}
	TAU	37.66 ± 1.3^{a}	52.66 ± 3.5^{b}	32.93 ± 2.3^{a}	40.48 ± 2.5^{a}
**Fe (ppm)**	UCD	37.66 ± 0.9^{a}	38.32 ± 1.3^{a}	28.88 ± 0.4^{b}	29.83 ± 1.7^{b}
	TAU	31.24 ± 1.6^{b}	34.24 ± 1.3^{a}	25.48 ± 1.4^{c}	24.73 ± 1.6^{c}
**Cu (ppm)**	UCD	5.82 ± 0.1^{a}	8.62 ± 2.5^{a}	5.72 ± 0.2^{a}	5.49 ± 0.3^{a}
	TAU	6.99 ± 0.1^{a}	6.7 ± 0.2^{a}	6.18 ± 0.1^{b}	4.83 ± 0.2^{c}

Table S2. Summary of raw and trimmed Illumina sequencing reads and mapping rates from each biological replicate.

Time point	Genotype	Replicate	Raw reads	Trimmed reads	% mapped to defined loci	% uniquely mapped to defined loci
**Heading**	**WT**	1	38,665,098	34,773,202	93.99	58.74
		2	39,475,711	39,297,400	94.15	59.59
		3	31,381,725	29,301,745	94.24	58.99
		4	33,848,760	33,264,328	93.36	58.36
	gpc-A1	1	32,102,685	28,868,068	94.35	58.83
		2	37,154,487	36,992,899	94.49	59.75
		3	31,889,453	29,763,897	94.52	58.80
		4	30,140,634	29,622,936	93.65	58.34
	gpc-A1/gpc-B2	1	41,424,154	37,211,906	94.65	52.42
		2	39,970,397	39,787,853	93.83	59.02
		3	33,530,071	31,320,928	93.64	58.27
		4	32,944,098	32,368,503	93.34	58.03
**12 DAA**	**WT**	1	39,834,397	35,863,953	93.23	58.44
		2	40,040,076	39,856,173	93.72	59.60
		3	34,534,155	32,265,656	93.00	57.59
		4	85,099,084	83,638,792	92.69	58.57
	gpc-A1	1	35,410,326	31,884,963	94.65	49.51
		2	39,337,625	39,151,166	93.36	59.22
		3	33,414,297	31,248,455	93.48	59.11
		4	32,263,546	31,713,135	92.51	58.12
	gpc-A1/gpc-B2	1	31,076,406	27,987,586	93.97	58.98
		2	35,012,331	34,832,872	91.94	58.38
		3	25,188,810	23,538,518	93.25	58.44
		4	28,255,874	27,763,518	93.85	59.00
**22 DAA**	**WT**	1	40,666,581	36,539,348	92.83	58.82
		2	37,814,738	37,635,671	92.18	59.44
		3	33,437,511	31,252,956	92.87	59.07
		4	31,528,730	30,988,194	93.16	59.17
	gpc-A1	1	37,399,266	33,359,639	92.56	59.56
		2	38,746,000	38,565,077	91.79	59.77
		3	28,514,156	26,523,981	93.75	59.41
		4	30,074,410	29,535,381	93.73	59.68
	gpc-A1/gpc-B2	1	45,208,215	40,694,293	93.20	58.49
		2	39,929,531	39,736,903	91.96	59.29
		3	36,835,190	34,436,035	93.17	59.44
		4	33,732,441	33,162,000	93.48	58.34
	**Average**		36,552,249	34,854,109	93.40	58.46

Table S3. 26 genes commonly differentially regulated during senescence between the current study and Gregersen & Holm (2007).

Probe name (Gregersen et al. 2011)	Corresponding URGI contig	BLAST e-value	Hit description
G05_p234_plate_12	6BL-ab-k71_contig_4275858:15270-21181	0	gi|1657845|gb|AAB18202.1| cold acclimation protein WCOR410c [Triticum aestivum]
H01_p436_plate_12	4BL-ab-k71_contig_6973512:3778-8889	5E-84	gi|146231063|gb|ABQ12768.1| hypersensitive response protein [Triticum aestivum]
C04_p638_plate_2	2AS-ab-k71_contig_5252955:1-993	0	gi|474253786|gb|EMS60319.1| Ribonuclease 1 [Triticum urartu]
B10_p234_plate_26	3AL-ab-k71_contig_4355214:1431-1859	2E-64	-
D06_p335_plate_3	1BL-ab-k71_contig_3863282:2209-5503	5E-65	gi|475538858|gb|EMT09311.1| Aldehyde dehydrogenase family 2 member C4 [Aegilops tauschii]
A04_a22_plate_12	2AL-ab-k71_contig_6315374:6012-9120	1E-120	gi|475572136|gb|EMT16425.1| Cysteine proteinase 1 [Aegilops tauschii]
D02_j223_plate_15	2AL-ab-k71_contig_6399751:5443-12037	1E-169	gi|475609973|gb|EMT27565.1| ABC transporter C family member 9 [Aegilops tauschii]
A02_e411_plate_2	6BL-ab-k71_contig_1176509:1-8128	5E-86	gi|299482520|gb|ADJ19186.1| lysine ketoglutarate reductase/saccharopine dehydrogenase [Triticum turgidum]
D10_e411_plate_13	1AL_v2-ab-k71_contig_3885764:9943-15907	1E-146	gi|460373787|ref|XP_004232695.1| PREDICTED: aspartic proteinase-like [Solanum lycopersicum]
F03_N130_plate_64	6AL-ab-k71_contig_5767722:1-2056	1E-120	gi|475621610|gb|EMT31542.1| 3-ketoacyl-CoA thiolase 2, peroxisomal [Aegilops tauschii]
G03_p234_plate_13	3B-ab-k71_contig_10644572:1675-3600	0	gi|474436565|gb|EMS68215.1| UDP-glycosyltransferase 87A1 [Triticum urartu]
C08_d26_plate_11	6BL-ab-k71_contig_4396688:584-4562	1E-68	gi|475574510|gb|EMT17003.1| Acetylornithine deacetylase [Aegilops tauschii]
B03_p234_plate_11_run_2	2AS-ab-k71_contig_5303733:4078-6463	0	gi|326493682|dbj|BAJ85302.1| predicted protein [Hordeum vulgare subsp. vulgare]
A11_a11_plate_3	4BS-ab-k71_contig_4861976:6480-7581	2E-36	gi|474290679|gb|EMS61268.1| Metallothionein-like protein 1 [Triticum urartu]
E01_N130_plate_48	5BL-ab-k71_contig_10818068:1381-4394	0	gi|357122996|ref|XP_003563199.1| PREDICTED: phospholipase C 4-like [Brachypodium distachyon]
B01_p436_plate_8_run_2	2AS-ab-k71_contig_5256148:581-5506	4E-51	gi|357116071|ref|XP_003559808.1| PREDICTED: acyl-CoA dehydrogenase family member 10-like [Brachypodium distachyon]
C02_p335_plate_7	7BS-ab-k71_contig_584435:1-200	1E-99	gi|475625512|gb|EMT32999.1| NAC domain-containing protein 18 [Aegilops tauschii]
B03_h116_plate_2	4BL-ab-k71_contig_7000160:3976-8945	3E-69	gi|475530866|gb|EMT07974.1| Prolyl 3-hydroxylase 1 [Aegilops tauschii]
D08_n129_plate_9	1AS-ab-k71_contig_310232:674-1526	4E-99	-
H07_p436_plate_10	4AL_v2-ab-k71_contig_4AL_7112091:4128-8300	1E-170	gi|475509418|gb|EMT05187.1| Glutamate decarboxylase 1 [Aegilops tauschii]
E06_p335_plate_6	5BS-ab-k71_contig_2277410:2149-11495	1E-126	gi|283970976|gb|ADB54814.1| universal stress protein 9303 [Hordeum vulgare subsp. vulgare] gi|283970978|gb|ADB54815.1| universal stress protein 9303 [Hordeum vulgare subsp. vulgare]
H08_p234_plate_14	1BL-ab-k71_contig_3868206:2733-3775	0	gi|473939569|gb|EMS50260.1| Ferredoxin-6, chloroplastic [Triticum urartu]
C05_l226_plate_3	2AL-ab-k71_contig_6340847:3105-5951	4E-31	gi|4325045|gb|AAD17232.1| S-adenosylmethionine decarboxylase precursor [Triticum aestivum]
E09_p335_plate_3	1AS-ab-k71_contig_2470305:166-1376	3E-72	gi|473881465|gb|EMS47979.1| putative glutathione S-transferase GSTF1 [Triticum urartu]
D09_p335_plate_7	3AL-ab-k71_contig_4427563:4889-5726	0	gi|514830677|gb|AGO59325.1| MYB13 transcription factor [Triticum aestivum]
D10_p537_plate_6	3B-ab-k71_contig_10631124:2445-5741	2E-45	gi|475497046|gb|EMT03967.1| GDSL esterase/lipase [Aegilops tauschii]

Table S4. 196 affymetrix contigs from Jukanti et al. 2008 also differentially expressed during senescence.

Affymetrix ID (Jukanti et al. 2008)	Corresponding URGI contig	BLAST e-value	Hit description
Contig903_at	5BL-ab-k71_contig_10815975:7698-9613	1.00E-154	gi|474396172|gb|EMS65694.1| Alcohol dehydrogenase 2 [Triticum urartu]
HF12K17r_at	2AS-ab-k71_contig_5273012:812-7972	8.00E-80	gi|326492077|dbj|BAJ98263.1| predicted protein [Hordeum vulgare subsp. vulgare]
Contig24024_at	6AL-ab-k71_contig_5814401:2506-3427	2.00E-15	gi|326502074|dbj|BAK06529.1| predicted protein [Hordeum vulgare subsp. vulgare]
Contig14921_s_at	3B-ab-k71_contig_10604827:215-5360	4.00E-78	gi|474066737|gb|EMS54176.1| Purple acid phosphatase 2 [Triticum urartu]
Contig16604_at	4BS-ab-k71_contig_4901464:24-823	1E-147	#N/A
Contig15056_at	2BL-ab-k71_contig_7971302:3009-7466	9E-77	gi|326512690|dbj|BAK03252.1| predicted protein [Hordeum vulgare subsp. vulgare]
Contig7857_s_at	2AS-ab-k71_contig_5225988:1-3104	1E-125	gi|326528587|dbj|BAJ93475.1| predicted protein [Hordeum vulgare subsp. vulgare]
Contig1402_at	3AS-ab-k71_contig_2573541:1-247	3E-38	gi|475601114|gb|EMT24719.1| GDSL esterase/lipase [Aegilops tauschii]
HW08K02u_at	4BS-ab-k71_contig_4865249:5225-6279	1E-59	gi|326514238|dbj|BAJ92269.1| predicted protein [Hordeum vulgare subsp. vulgare]
HV10A20u_s_at	1AL_v2-ab-k71_contig_3912549:1123-3112	4E-16	gi|474392822|gb|EMS65496.1| Cysteine proteinase RD21a [Triticum urartu]
Contig21245_at	3B-ab-k71_contig_10417977:695-3511	0	gi|326494096|dbj|BAJ85510.1| predicted protein [Hordeum vulgare subsp. vulgare]
Contig26_at	5BL-ab-k71_contig_10806933:541-1198	1E-178	gi|195617694|gb|ACG30677.1| histone H4 [Zea mays]
Contig7112_at	4AL_v2-ab-k71_contig_4AL_7160884:3225-4267	4E-64	gi|157312498|gb|ABV32166.1| UFG2 [Triticum aestivum]
Contig7433_at	2AL-ab-k71_contig_6370536:4028-8220	1E-119	gi|474154143|gb|EMS57069.1| Abscisic acid-inducible protein kinase [Triticum urartu]
Contig7433_s_at	2AL-ab-k71_contig_6370536:4028-8220	1E-119	gi|474154143|gb|EMS57069.1| Abscisic acid-inducible protein kinase [Triticum urartu]
Contig19290_at	2BL-ab-k71_contig_7936650:1-1358	0	gi|474268501|gb|EMS60789.1| UDP-glycosyltransferase 73C6 [Triticum urartu]
Contig22948_at	4BL-ab-k71_contig_6856755:3224-5930	2E-52	gi|326528067|dbj|BAJ89085.1| predicted protein [Hordeum vulgare subsp. vulgare]
rbaal12n12_s_at	5BL-ab-k71_contig_10856642:7311-10619	1E-158	gi|475574369|gb|EMT16972.1| Putative transporter [Aegilops tauschii]
Contig3935_s_at	7BL-ab-k71_contig_6509502:9-3078	4E-54	gi|473936597|gb|EMS50148.1| Reticulon-like protein B2 [Triticum urartu]
HS06C20u_s_at	7BL-ab-k71_contig_6509502:9-3078	3E-88	gi|473936597|gb|EMS50148.1| Reticulon-like protein B2 [Triticum urartu]
Contig7686_at	1BL-ab-k71_contig_3825950:1469-5204	0	gi|56409844|emb|CAI30070.1| glycosyltransferase [Triticum aestivum]
Contig20207_s_at	3AL-ab-k71_contig_1034593:1-6300	4E-72	gi|326504974|dbj|BAJ99498.1| predicted protein [Hordeum vulgare subsp. vulgare]
Contig7343_s_at	2AL-ab-k71_contig_6433511:6743-7054	1E-24	gi|326516112|dbj|BAJ88079.1| predicted protein [Hordeum vulgare subsp. vulgare]
Contig14595_at	5BL-ab-k71_contig_10872426:20719-23310	0	#N/A
HS06J22u_s_at	1AL_v2-ab-k71_contig_3897631:1-3252	6E-99	gi|326488911|dbj|BAJ98067.1| predicted protein [Hordeum vulgare subsp. vulgare] gi|326506402|dbj|BAJ86519.1| predicted protein [Hordeum vulgare subsp. vulgare]
Contig9006_at	1AL_v2-ab-k71_contig_3912549:1123-3112	0	gi|474392822|gb|EMS65496.1| Cysteine proteinase RD21a [Triticum urartu]
Contig9352_at	3AS-ab-k71_contig_3442141:693-1706	0	gi|383100757|emb|CCG47989.1| glycosyltransferase, HGA-like, expressed [Triticum aestivum]
EBpi01_SQ002_N08_at	4AL_v2-ab-k71_contig_4AL_7155714:1227-3637	9E-20	gi|326503002|dbj|BAJ99126.1| predicted protein [Hordeum vulgare subsp. vulgare]
Contig9110_at	7BS-ab-k71_contig_3151221:1-523	1E-104	gi|326489587|dbj|BAK01774.1| predicted protein [Hordeum vulgare subsp. vulgare] gi|326512308|dbj|BAJ99509.1| predicted protein [Hordeum vulgare subsp. vulgare] gi|326529319|dbj|BAK01053.1| predicted protein [Hordeum vulgare subsp. vulgare]
HV_CEa0011J22r2_at	3AL-ab-k71_contig_4345443:1-775	1E-41	gi|474224339|gb|EMS59376.1| hypothetical protein TRIUR3_02071 [Triticum urartu]

Contig18428_at	3AL-ab-k71_contig_1034593:1-6300	0	gi|326504974|dbj|BAJ99498.1| predicted protein [Hordeum vulgare subsp. vulgare]
Contig9351_s_at	3AS-ab-k71_contig_3442141:693-1706	0	gi|383100757|emb|CCG47989.1| glycosyltransferase, HGA-like, expressed [Triticum aestivum]
HT12G07u_s_at	6AL-ab-k71_contig_5818539:1-7046	3E-09	gi|356463714|gb|AET08895.1| vacuolar processing enzyme 2 [Aegilops tauschii]
Contig17964_at	4AS_v2-ab-k71_contig_5977931:3675-4720	0	gi|326494882|dbj|BAJ94560.1| predicted protein [Hordeum vulgare subsp. vulgare]
Contig13385_s_at	2BL-ab-k71_contig_8062334:2765-3987	1E-117	gi|326530163|dbj|BAK08361.1| predicted protein [Hordeum vulgare subsp. vulgare]
Contig20405_at	3B-ab-k71_contig_10615303:1399-4851	1E-103	gi|326500940|dbj|BAJ95136.1| predicted protein [Hordeum vulgare subsp. vulgare]
Contig7359_at	4BL-ab-k71_contig_6887705:1-1034	1E-161	gi|475595559|gb|EMT23077.1| Pheophorbide a oxygenase, chloroplastic [Aegilops tauschii]
Contig7503_at	1AL_v2-ab-k71_contig_3960754:94-4380	5E-68	gi|326523819|dbj|BAJ93080.1| predicted protein [Hordeum vulgare subsp. vulgare] gi|326531656|dbj|BAJ97832.1| predicted protein [Hordeum vulgare subsp. vulgare]
Contig3892_at	2BL-ab-k71_contig_8088049:357-4123	0	gi|475588310|gb|EMT20813.1| Putative quinone-oxidoreductase-like protein, chloroplastic [Aegilops tauschii]
Contig5654_at	2AS-ab-k71_contig_5225155:1786-5351	1E-174	gi|345447290|gb|AEN92260.1| arginase [Triticum aestivum]
HA18O08r_s_at	5AL-ab-k95_contig_1128960:517-4580	2E-64	gi|474114511|gb|EMS55823.1| putative ornithine aminotransferase [Triticum urartu]
Contig11129_at	2BS-ab-k71_contig_5194333:8297-9616	1E-107	gi|475510892|gb|EMT05328.1| Adipocyte plasma membrane-associated protein [Aegilops tauschii]
Contig2736_s_at	5AL-ab-k95_contig_2809387:2645-5504	1E-127	gi|473891753|gb|EMS48763.1| Beta-glucosidase 31 [Triticum urartu]
Contig3691_at	2AS-ab-k71_contig_5252955:1-993	0	gi|474253786|gb|EMS60319.1| Ribonuclease 1 [Triticum urartu]
Contig7997_at	6BL-ab-k71_contig_3771316:67-1562	3E-62	gi|326513484|dbj|BAJ92093.1| predicted protein [Hordeum vulgare subsp. vulgare] gi|326514820|dbj|BAJ99771.1| predicted protein [Hordeum vulgare subsp. vulgare] gi|326516984|dbj|BAJ96484.1| predicted protein [Hordeum vulgare subsp. vulgare] gi|326525907|dbj|BAJ93130.1| predicted protein [Hordeum vulgare subsp. vulgare]
Contig6152_at	7BS-ab-k71_contig_3097754:654-17033	1E-155	gi|475589772|gb|EMT21266.1| ABC transporter C family member 10 [Aegilops tauschii]
HV_CEb0005N09r2_at	6AS-ab-k71_contig_4359475:3564-5827	3E-85	gi|474237122|gb|EMS59633.1| Epoxide hydrolase 2 [Triticum urartu]
HVSMEm0007B06r2_s_at	5AL-ab-k95_contig_22698:1-1478	1E-132	gi|474167057|gb|EMS57469.1| KDEL-tailed cysteine endopeptidase CEP1 [Triticum urartu]
HV_CEa0006M06r2_s_at	4AL_v2-ab-k71_contig_4AL_7075859:3208-6993	1E-59	gi|326524432|dbj|BAK00599.1| predicted protein [Hordeum vulgare subsp. vulgare]
Contig10193_at	3AL-ab-k71_contig_4336869:1-1893	1E-69	gi|474213532|gb|EMS58952.1| Elicitor-responsive protein 1 [Triticum urartu]
Contig23092_at	7BS-ab-k71_contig_3088434:3365-7082	0	gi|326522618|dbj|BAK07771.1| predicted protein [Hordeum vulgare subsp. vulgare]
Contig10270_at	5AL-ab-k95_contig_2783071:1-1528	0	gi|326505554|dbj|BAJ95448.1| predicted protein [Hordeum vulgare subsp. vulgare] gi|326515132|dbj|BAK03479.1| predicted protein [Hordeum vulgare subsp. vulgare]
Contig24230_at	2AS-ab-k71_contig_5199858:1-856	1E-134	gi|348549744|emb|CBZ41151.1| NAC transcription factor [Hordeum vulgare subsp. vulgare]
Contig17818_at	1AS-ab-k71_contig_3288158:270-2003	0	gi|326518772|dbj|BAJ92547.1| predicted protein [Hordeum vulgare subsp. vulgare]
HV_CEa0006E02r2_s_at	6AL-ab-k71_contig_5830900:430-3997	2E-77	gi|473922892|gb|EMS49728.1| Quinone oxidoreductase PIG3 [Triticum urartu]
Contig7293_at	2BL-ab-k71_contig_8052619:2011-6240	0	gi|326519885|dbj|BAK03867.1| predicted protein [Hordeum vulgare subsp. vulgare]
Contig17276_at	5BL-ab-k71_contig_348262:1118-7118	0	gi|357153424|ref|XP_003576447.1| PREDICTED: ABC transporter A family member 7-like [Brachypodium distachyon]
Contig20144_s_at	4AL_v2-ab-k71_contig_4AL_7155714:1227-3637	2E-23	gi|326503002|dbj|BAJ99126.1| predicted protein [Hordeum vulgare subsp. vulgare]
Contig8056_at	6AL-ab-k71_contig_5760608:1-612	7E-78	gi|474065824|gb|EMS54111.1| Peroxisomal multifunctional enzyme type 2 [Triticum urartu]

			gi|475624481|gb|EMT32631.1| Peroxisomal multifunctional enzyme type 2 [Aegilops tauschii]
Contig13658_at	2BS-ab-k71_contig_5208331:5065-7158	0	gi|371924997|tpe|CBZ41159.1| TPA: NAC transcription factor [Hordeum vulgare subsp. vulgare]
Contig13046_at	1BL-ab-k71_contig_3904034:661-2554	2E-62	gi|326520874|dbj|BAJ92800.1| predicted protein [Hordeum vulgare subsp. vulgare]
			gi|326531956|dbj|BAK01354.1| predicted protein [Hordeum vulgare subsp. vulgare]
Contig10594_at	7BS-ab-k71_contig_909259:1-1586	0	gi|475558485|gb|EMT13108.1| hypothetical protein F775_27330 [Aegilops tauschii]
Contig5299_at	1BL-ab-k71_contig_3868206:2733-3775	0	gi|473939569|gb|EMS50260.1| Ferredoxin-6, chloroplastic [Triticum urartu]
Contig10981_at	5BS-ab-k71_contig_2274553:453-5698	1E-112	gi|357157153|ref|XP_003577703.1| PREDICTED: uncharacterized hydrolase HI_0588-like [Brachypodium distachyon]
Contig24105_at	4AS_v2-ab-k71_contig_5952279:5675-11064	1E-173	gi|326506432|dbj|BAJ86534.1| predicted protein [Hordeum vulgare subsp. vulgare]
Contig1403_at	3AS-ab-k71_contig_2573541:1-247	2E-67	gi|475601114|gb|EMT24719.1| GDSL esterase/lipase [Aegilops tauschii]
Contig11890_at	6AL-ab-k71_contig_5830900:430-3997	3E-96	gi|473922892|gb|EMS49728.1| Quinone oxidoreductase PIG3 [Triticum urartu]
Contig20575_at	1BL-ab-k71_contig_3894079:1-345	3E-83	gi|326512068|dbj|BAJ96015.1| predicted protein [Hordeum vulgare subsp. vulgare]
Contig9954_at	6BL-ab-k71_contig_4309090:343-4524	1E-132	gi|326496777|dbj|BAJ98415.1| predicted protein [Hordeum vulgare subsp. vulgare]
Contig6343_at	3B-ab-k71_contig_10520391:1337-6837	0	gi|326505418|dbj|BAJ95380.1| predicted protein [Hordeum vulgare subsp. vulgare]
Contig1404_at	3AS-ab-k71_contig_2573541:1-247	5E-80	gi|475601114|gb|EMT24719.1| GDSL esterase/lipase [Aegilops tauschii]
EBma03_SQ002_F16_s_at	4AS_v2-ab-k71_contig_5955108:4662-6252	6E-26	gi|326487554|dbj|BAK05449.1| predicted protein [Hordeum vulgare subsp. vulgare]
rbaal10h14_at	2BL-ab-k71_contig_8072661:19233-21176	3E-73	gi|326490093|dbj|BAJ94120.1| predicted protein [Hordeum vulgare subsp. vulgare]
			gi|326516896|dbj|BAJ96440.1| predicted protein [Hordeum vulgare subsp. vulgare]
Contig1404_x_at	3AS-ab-k71_contig_2573541:1-247	5E-80	gi|475601114|gb|EMT24719.1| GDSL esterase/lipase [Aegilops tauschii]
Contig15869_at	4AS_v2-ab-k71_contig_352920:1089-3423	0	gi|189172063|gb|ACD80387.1| WRKY40 transcription factor, partial [Triticum aestivum]
Contig10700_at	3B-ab-k71_contig_10699811:1993-2563	1E-91	gi|326490638|dbj|BAJ89986.1| predicted protein [Hordeum vulgare subsp. vulgare]
Contig4096_at	1BS-ab-k71_contig_3454525:1-7236	1E-135	gi|326498703|dbj|BAK02337.1| predicted protein [Hordeum vulgare subsp. vulgare]
Contig5177_s_at	1BL-ab-k71_contig_3830761:5135-10857	1E-162	gi|474317121|gb|EMS62134.1| Actin-7 [Triticum urartu]
			gi|475561024|gb|EMT13696.1| Actin-7 [Aegilops tauschii]
Contig4639_s_at	2AL-ab-k71_contig_6356061:2883-4074	6E-91	gi|474390725|gb|EMS65281.1| hypothetical protein TRIUR3_32617 [Triticum urartu]
Contig6008_s_at	1AL_v2-ab-k71_contig_3904007:1-2701	1E-171	gi|475600098|gb|EMT24434.1| hypothetical protein F775_32163 [Aegilops tauschii]
Contig9392_at	1BS-ab-k71_contig_3431786:6994-7732	2E-37	gi|475514050|gb|EMT05681.1| Low temperature-induced protein [Aegilops tauschii]
Contig26053_at	3AL-ab-k71_contig_4407808:2194-3672	7E-74	gi|475625209|gb|EMT32886.1| Jasmonate O-methyltransferase [Aegilops tauschii]
HV_CEa0008J10r2_s_at	3AS-ab-k71_contig_3379776:1910-2481	4E-20	#N/A
Contig2750_s_at	6AL-ab-k71_contig_5767722:1-2056	2E-55	gi|475621610|gb|EMT31542.1| 3-ketoacyl-CoA thiolase 2, peroxisomal [Aegilops tauschii]
Contig16179_s_at	1BS-ab-k71_contig_3423601:17002-26931	2E-13	gi|357130187|ref|XP_003566732.1| PREDICTED: phosphatidylinositol 3-kinase, root isoform-like [Brachypodium distachyon]
HVSMEl0013E16r2_s_at	6AL-ab-k71_contig_5791715:1-1294	4E-57	gi|326528223|dbj|BAJ93293.1| predicted protein [Hordeum vulgare subsp. vulgare]
			gi|326528473|dbj|BAJ93418.1| predicted protein [Hordeum vulgare subsp. vulgare]
Contig6381_at	3AL-ab-k71_contig_96597:80-1495	1E-137	gi|473874636|gb|EMS47816.1| Aldehyde dehydrogenase family 2 member C4 [Triticum urartu]
Contig16535_at	5BS-ab-k71_contig_2266199:109-6401	1E-115	gi|357161700|ref|XP_003579177.1| PREDICTED: methylcrotonoyl-CoA carboxylase subunit alpha, mitochondrial-like [Brachypodium distachyon]
HW02O09u_s_at	5AL-ab-k95_contig_565469:1301-3254	6E-60	gi|474265415|gb|EMS60690.1| Auxin-induced protein

			5NG4 [Triticum urartu]
rbaal23d16_s_at	3AL-ab-k71_contig_4332102:492-3349	5E-28	gi|475567132|gb|EMT15199.1| putative Isocitrate dehydrogenase (NADP), chloroplastic [Aegilops tauschii]
HVSMEl0010P10f_x_at	2BS-ab-k71_contig_5201747:10374-13960	8E-35	gi|345447290|gb|AEN92260.1| arginase [Triticum aestivum]
Contig6659_s_at	3B-ab-k71_contig_10639283:103-4231	1E-102	gi|474156727|gb|EMS57131.1| Sphingosine-1-phosphate lyase [Triticum urartu]
Contig2253_s_at	2AL-ab-k71_contig_6439401:36-885	0	gi|475515312|gb|EMT05831.1| Histone H1 [Aegilops tauschii]
Contig10984_at	3AL-ab-k71_contig_4450714:278-8391	0	gi|161702907|gb|ABX76295.1| neutral ceramidase [Triticum aestivum]
Contig25897_at	7BS-ab-k71_contig_3065267:1-5116	0	gi|474036949|gb|EMS53193.1| FACT complex subunit SPT16 [Triticum urartu]
rbaal21e22_x_at	6AL-ab-k71_contig_5767722:1-2056	3E-18	gi|475621610|gb|EMT31542.1| 3-ketoacyl-CoA thiolase 2, peroxisomal [Aegilops tauschii]
HV_CEa0008J10r2_at	3AS-ab-k71_contig_3379776:1910-2481	4E-20	#N/A
Contig17260_at	6AS-ab-k71_contig_4351215:1-1669	9E-99	gi|326497895|dbj|BAJ94810.1| predicted protein [Hordeum vulgare subsp. vulgare] gi|326531478|dbj|BAJ97743.1| predicted protein [Hordeum vulgare subsp. vulgare]
Contig17135_s_at	2BL-ab-k71_contig_8062334:2765-3987	3E-44	gi|326530163|dbj|BAK08361.1| predicted protein [Hordeum vulgare subsp. vulgare]
EBpi03_SQ001_I14_at	4AL_v2-ab-k71_contig_4AL_7099893:6436-10911	8E-20	gi|326511956|dbj|BAJ95959.1| predicted protein [Hordeum vulgare subsp. vulgare]
Contig617_x_at	6AS-ab-k71_contig_4349651:4661-5719	0	gi|475604137|gb|EMT25652.1| hypothetical protein F775_43704 [Aegilops tauschii]
Contig433_x_at	6BS-ab-k71_contig_2935443:1-457	0	gi|31753114|gb|AAH53854.1| Unknown (protein for IMAGE:5194336), partial [Homo sapiens] gi|111494016|gb|AAI11385.1| Unknown (protein for IMAGE:5198300), partial [Homo sapiens]
Contig346_at	5AL-ab-k95_contig_2799832:1371-2762	7E-91	gi|4090293|emb|CAA10497.1| hypothetical protein [Secale cereale]
Contig6411_at	4AS_v2-ab-k71_contig_5994312:6000-8002	0	gi|475605702|gb|EMT26171.1| 3-ketoacyl-CoA synthase 1 [Aegilops tauschii]
Contig17360_at	6AL-ab-k71_contig_5757005:1129-7237	7E-62	gi|357145196|ref|XP_003573558.1| PREDICTED: methionine aminopeptidase 1B, chloroplastic-like [Brachypodium distachyon]
Contig13068_at	3B-ab-k71_contig_10402369:1602-2690	3E-43	#N/A
Contig16209_at	3B-ab-k71_contig_10690339:4659-6750	1E-58	gi|475580681|gb|EMT18662.1| Glucoamylase [Aegilops tauschii]
Contig11332_at	3B-ab-k71_contig_10730106:2887-7639	9E-96	gi|475615860|gb|EMT29587.1| Putative membrane protein [Aegilops tauschii]
Contig14144_at	6AL-ab-k71_contig_5764148:7642-9513	0	gi|473777884|gb|EMS46019.1| hypothetical protein TRIUR3_07308 [Triticum urartu]
HVSMEh0102G02f_x_at	4AS_v2-ab-k71_contig_5945881:18664-19150	2E-29	gi|326506444|dbj|BAJ86540.1| predicted protein [Hordeum vulgare subsp. vulgare]
Contig21024_at	4AS_v2-ab-k71_contig_5970292:5240-10627	1E-108	gi|254885381|emb|CBA11528.1| sulphate transporter [Triticum aestivum]
Contig16113_at	2AL-ab-k71_contig_6364940:5698-6948	0	gi|326520605|dbj|BAK07561.1| predicted protein [Hordeum vulgare subsp. vulgare]
Contig2985_s_at	2BS-ab-k71_contig_5231013:12845-20601	0	gi|14861035|gb|AAK72401.1| Mg-chelatase subunit XANTHA-F [Hordeum vulgare subsp. vulgare]
Contig12563_s_at	6AS-ab-k71_contig_4385322:6110-7798	1E-126	gi|326528365|dbj|BAJ93364.1| predicted protein [Hordeum vulgare subsp. vulgare]
Contig16015_at	5BS-ab-k71_contig_2287895:5743-10850	1E-146	gi|475534641|gb|EMT08606.1| Coleoptile phototropism protein 1 [Aegilops tauschii]
Contig2433_s_at	4AL_v2-ab-k71_contig_4AL_7151445:3859-11701	1E-161	gi|474074549|gb|EMS54507.1| Acid phosphatase 1 [Triticum urartu]
Contig2111_s_at	2AL-ab-k71_contig_6359939:14373-15478	6E-77	gi|326521510|dbj|BAK00331.1| predicted protein [Hordeum vulgare subsp. vulgare]
Contig19029_at	7AL-ab-k71_contig_4470746:4134-4406	3E-67	gi|475577656|gb|EMT17876.1| Beta-galactosidase [Aegilops tauschii]
HA14G19r_at	5AL-ab-k95_contig_13914:1-3260	2E-72	gi|474343675|gb|EMS62751.1| 2-hydroxy-6-oxo-6-phenylhexa-2,4-dienoate hydrolase [Triticum urartu]
X12735_at	1AL_v2-ab-k71_contig_3715806:2709-4239	1E-131	gi|474060351|gb|EMS53958.1| Chlorophyll a-b binding protein 3C, chloroplastic [Triticum urartu]
Contig13735_at	6BL-ab-k71_contig_127065:3397-4210	1E-125	gi|326488427|dbj|BAJ93882.1| predicted protein [Hordeum vulgare subsp. vulgare]
Contig21274_at	4AL_v2-ab-k71_contig_4AL_7125345:8254-15574	1E-138	gi|326492676|dbj|BAJ90194.1| predicted protein [Hordeum vulgare subsp. vulgare]

Contig820_at	4AL_v2-ab-k71_contig_4AL_5754445:1016-3177	0	gi|473913339|gb|EMS49688.1| Chlorophyll a-b binding protein CP26, chloroplastic [Triticum urartu]
HV_CEa0016F11f_at	4BL-ab-k71_contig_6992715:1-4024	6E-14	gi|357113513|ref|XP_003558547.1| PREDICTED: uncharacterized protein LOC100823410 [Brachypodium distachyon]
Contig16020_at	4BS-ab-k71_contig_4937574:115-7508	1E-124	gi|357156916|ref|XP_003577619.1| PREDICTED: uncharacterized protein LOC100836694 [Brachypodium distachyon]
Contig2412_at	2AS-ab-k71_contig_5190107:3940-11214	1E-161	gi|357122526|ref|XP_003562966.1| PREDICTED: uncharacterized protein LOC100831317 [Brachypodium distachyon]
Contig3396_s_at	3B-ab-k71_contig_10628326:166-917	1E-179	gi|475559550|gb|EMT13370.1| hypothetical protein F775_28691 [Aegilops tauschii]
EBro07_SQ002_K15_x_at	4BL-ab-k71_contig_7034718:16725-18998	4E-26	#N/A
Contig194_s_at	5AL-ab-k95_contig_2774278:3264-4547	0	gi|475614191|gb|EMT29003.1| Chlorophyll a-b binding protein, chloroplastic [Aegilops tauschii]
HVSMEn0020J05f_x_at	5BL-ab-k71_contig_10916028:1304-6876	1E-108	gi|326513480|dbj|BAK06980.1| predicted protein [Hordeum vulgare subsp. vulgare]
HVSMEh0080N02r2_at	2AS-ab-k71_contig_5263481:3990-5197	3E-25	gi|400879|sp|P31093.1|PSAN_HORVU RecName: Full=Photosystem I reaction center subunit N, chloroplastic; Short=PSI-N; Flags: Precursor gi|19095|emb|CAA47056.1| photosystem I subunit N [Hordeum vulgare subsp. vulgare] gi|326517280|dbj|BAK00007.1| predicted protein [Hordeum vulgare subsp. vulgare]
baak46p14_s_at	3AS-ab-k71_contig_3334610:1-2284	2E-53	gi|58339283|gb|AAW66004.1| leucine zipper protein zip1 [Triticum aestivum]
HVSMEi0005B04r2_x_at	2BS-ab-k71_contig_3595733:4298-5840	2E-16	gi|326528195|dbj|BAJ89149.1| predicted protein [Hordeum vulgare subsp. vulgare]
Contig497_s_at	5AL-ab-k95_contig_2799832:1371-2762	3E-64	gi|4090293|emb|CAA10497.1| hypothetical protein [Secale cereale]
Contig3183_at	6BL-ab-k71_contig_2539176:1-221	8E-55	gi|475559300|gb|EMT13311.1| Geranylgeranyl diphosphate reductase, chloroplastic [Aegilops tauschii]
Contig14542_at	3B-ab-k71_contig_10550500:5495-21268	1E-131	gi|475570720|gb|EMT16094.1| Alpha-amylase [Aegilops tauschii]
Contig4700_at	3B-ab-k71_contig_10631745:24-4235	0	gi|357141239|ref|XP_003572147.1| PREDICTED: uncharacterized protein At4g01050, chloroplastic-like [Brachypodium distachyon]
Contig3146_at	1AS-ab-k71_contig_2183291:104-3156	0	gi|354681874|dbj|BAL04917.1| glutamyl tRNA reductase [Triticum aestivum]
HV_CEa0015D14r2_at	5BL-ab-k71_contig_10902908:2670-5635	1E-176	gi|326531042|dbj|BAK04872.1| predicted protein [Hordeum vulgare subsp. vulgare]
Contig5026_at	6AS-ab-k71_contig_3291344:4049-4863	9E-83	gi|475596216|gb|EMT23305.1| hypothetical protein F775_30333 [Aegilops tauschii]
Contig23482_at	6AL-ab-k71_contig_5826035:1-3331	1E-55	gi|326502018|dbj|BAK06501.1| predicted protein [Hordeum vulgare subsp. vulgare]
HV_CEb0017F03r2_s_at	7AS-ab-k71_contig_4215480:1887-3348	1E-144	gi|225690802|gb|ACO06087.1| chlorophyll a-b binding protein [Triticum aestivum]
Contig14841_at	5BS-ab-k71_contig_2261475:1-2128	2E-74	gi|357114424|ref|XP_003559000.1| PREDICTED: uncharacterized protein LOC100832376 [Brachypodium distachyon]
Contig8679_at	5AL-ab-k95_contig_2748254:476-3293	1E-171	gi|326520313|dbj|BAK07415.1| predicted protein [Hordeum vulgare subsp. vulgare]
S78730_at	5BL-ab-k71_contig_10916028:1304-6876	1E-149	gi|326513480|dbj|BAK06980.1| predicted protein [Hordeum vulgare subsp. vulgare]
Contig7304_s_at	6BS-ab-k71_contig_3024079:10228-11218	0	gi|475458442|gb|EMT01926.1| O-methyltransferase ZRP4 [Aegilops tauschii]
Contig5659_at	7BS-ab-k71_contig_3068208:1-163	7E-77	gi|475558039|gb|EMT12976.1| 50S ribosomal protein 6, chloroplastic [Aegilops tauschii]
Contig20267_at	5BL-ab-k71_contig_10790778:15407-18782	1E-124	gi|326489459|dbj|BAK01710.1| predicted protein [Hordeum vulgare subsp. vulgare]
Contig9660_at	6AL-ab-k71_contig_5808587:152-3532	2E-63	gi|326494976|dbj|BAJ85583.1| predicted protein [Hordeum vulgare subsp. vulgare] gi|326506224|dbj|BAJ86430.1| predicted protein [Hordeum vulgare subsp. vulgare]
HVSMEh0080L10r2_x_at	7BS-ab-k71_contig_3164750:1-1240	1E-80	gi|225690802|gb|ACO06087.1| chlorophyll a-b binding protein [Triticum aestivum]
rbah53j14_at	2BL-ab-k71_contig_7993541:454-2439	5E-80	gi|326500100|dbj|BAJ90885.1| predicted protein [Hordeum vulgare subsp. vulgare] gi|326527749|dbj|BAK08149.1| predicted protein

			[Hordeum vulgare subsp. vulgare]
Contig1208_s_at	7BS-ab-k71_contig_3164750:1-1240	0	gi|225690802|gb|ACO06087.1| chlorophyll a-b binding protein [Triticum aestivum]
Contig7305_at	6BS-ab-k71_contig_3024079:10228-11218	0	gi|475458442|gb|EMT01926.1| O-methyltransferase ZRP4 [Aegilops tauschii]
Contig2766_s_at	1AL_v2-ab-k71_contig_3874670:3902-5985	1E-152	gi|10720236|sp|Q42850.1|PORB_HORVU RecName: Full=Protochlorophyllide reductase B, chloroplastic; Short=PCR B; AltName: Full=NADPH-protochlorophyllide oxidoreductase B; Short=POR B; Flags: Precursor gi|683476|emb|CAA59228.1| NADPH dehydrogenase [Hordeum vulgare] gi|326488707|dbj|BAJ97965.1| predicted protein [Hordeum vulgare subsp. vulgare] gi|326493324|dbj|BAJ85123.1| predicted protein [Hordeum vulgare subsp. vulgare] gi|326494290|dbj|BAJ90414.1| predicted protein [Hordeum vulgare subsp. vulgare]
Contig12574_at	2AL-ab-k71_contig_6437732:12404-19901	0	gi|474399175|gb|EMS65931.1| Lipoxygenase 1 [Triticum urartu]
EBed02_SQ002_E18_s_at	6AL-ab-k71_contig_5789067:4555-10598	3E-52	gi|218191232|gb|EEC73659.1| hypothetical protein OsI_08191 [Oryza sativa Indica Group]
Contig21126_at	4BL-ab-k71_contig_4768862:125-3354	2E-72	gi|154551059|gb|ABS83562.1| boron transporter [Hordeum vulgare] gi|154551061|gb|ABS83563.1| boron transporter [Hordeum vulgare] gi|160346960|gb|ABX26122.1| putative boron transporter 2 [Hordeum vulgare]
Contig8854_at	6AL-ab-k71_contig_5789067:4555-10598	1E-142	gi|218191232|gb|EEC73659.1| hypothetical protein OsI_08191 [Oryza sativa Indica Group]
HVSMEa0012N07r2_at	3B-ab-k71_contig_10594544:114-2512	4E-35	gi|326523645|dbj|BAJ92993.1| predicted protein [Hordeum vulgare subsp. vulgare] gi|326524189|dbj|BAJ97105.1| predicted protein [Hordeum vulgare subsp. vulgare]
Contig2762_at	1AL_v2-ab-k71_contig_3874670:3902-5985	0	gi|10720236|sp|Q42850.1|PORB_HORVU RecName: Full=Protochlorophyllide reductase B, chloroplastic; Short=PCR B; AltName: Full=NADPH-protochlorophyllide oxidoreductase B; Short=POR B; Flags: Precursor gi|683476|emb|CAA59228.1| NADPH dehydrogenase [Hordeum vulgare] gi|326488707|dbj|BAJ97965.1| predicted protein [Hordeum vulgare subsp. vulgare] gi|326493324|dbj|BAJ85123.1| predicted protein [Hordeum vulgare subsp. vulgare] gi|326494290|dbj|BAJ90414.1| predicted protein [Hordeum vulgare subsp. vulgare]
Contig3148_at	1BS-ab-k71_contig_3481555:1190-3041	0	gi|354681872|dbj|BAL04916.1| glutamyl tRNA reductase [Triticum aestivum]
Contig19438_at	2BL-ab-k71_contig_8039865:2256-6860	1E-175	gi|326489448|dbj|BAK01705.1| predicted protein [Hordeum vulgare subsp. vulgare]
Contig2177_at	3B-ab-k71_contig_10484319:1384-2702	0	gi|473789781|gb|EMS46316.1| Photosystem I reaction center subunit XI, chloroplastic [Triticum urartu]
rbah35f01_s_at	2AS-ab-k71_contig_5307368:5342-15094	6E-96	gi|326528371|dbj|BAJ93367.1| predicted protein [Hordeum vulgare subsp. vulgare]
Contig5059_s_at	1BL-ab-k71_contig_3797658:2212-4443	0	gi|475621673|gb|EMT31573.1| Ribonuclease 1 [Aegilops tauschii]
HVSMEf0013I09r2_at	2BS-ab-k71_contig_5166420:1548-4919	2E-46	#N/A
Contig9169_at	2BS-ab-k71_contig_5200682:6276-9820	0	gi|21668465|dbj|BAA07785.3| plastid omega-3 fatty acid desaturase [Triticum aestivum]
Contig628_x_at	5BL-ab-k71_contig_10831572:1917-3005	0	gi|475614190|gb|EMT29002.1| Chlorophyll a-b binding protein, chloroplastic [Aegilops tauschii]
Contig418_at	1BL-ab-k71_contig_1678368:429-2096	0	gi|475614190|gb|EMT29002.1| Chlorophyll a-b binding protein, chloroplastic [Aegilops tauschii]
Contig422_at	5AL-ab-k95_contig_2774278:3264-4547	0	gi|475614191|gb|EMT29003.1| Chlorophyll a-b binding protein, chloroplastic [Aegilops tauschii]
Contig841_x_at	1BL-ab-k71_contig_1678368:429-2096	0	gi|475614190|gb|EMT29002.1| Chlorophyll a-b binding protein, chloroplastic [Aegilops tauschii]
Contig828_s_at	1AL_v2-ab-k71_contig_3941328:9659-11114	0	gi|475614190|gb|EMT29002.1| Chlorophyll a-b binding protein, chloroplastic [Aegilops tauschii]
Contig14143_s_at	6AL-ab-k71_contig_5764148:7642-9513	1E-152	gi|473777884|gb|EMS46019.1| hypothetical protein TRIUR3_07308 [Triticum urartu]
Contig6_x_at	6AS-ab-k71_contig_4354575:1792-2909	0	gi|473952980|gb|EMS50796.1| Chlorophyll a-b binding

			protein, chloroplastic [Triticum urartu]
Contig11957_at	2BS-ab-k71_contig_5229550:15258-16617	0	gi|475518448|gb|EMT06200.1| Cytochrome P450 71C1 [Aegilops tauschii]
Contig347_s_at	6BS-ab-k71_contig_2981828:642-1842	0	gi|473952980|gb|EMS50796.1| Chlorophyll a-b binding protein, chloroplastic [Triticum urartu]
Contig6484_at	7AL-ab-k71_contig_4554300:1636-4345	0	gi|296044566|gb|ADG85703.1| putative NAC transcription factor [Triticum aestivum]
Contig2764_s_at	2BL-ab-k71_contig_7951021:10929-19547	0	gi|326507788|dbj|BAJ86637.1| predicted protein [Hordeum vulgare subsp. vulgare]
HV03G23u_s_at	1BL-ab-k71_contig_3792355:1-3151	2E-63	gi|354681870|dbj|BAL04915.1| glutamyl tRNA reductase [Triticum aestivum]
Contig636_at	1BL-ab-k71_contig_3813291:2683-4178	0	gi|475623771|gb|EMT32357.1| Chlorophyll a-b binding protein of LHCII type III, chloroplastic [Aegilops tauschii]
Contig12240_at	6BL-ab-k71_contig_4303428:96-1083	0	gi|473822355|gb|EMS47061.1| hypothetical protein TRIUR3_14995 [Triticum urartu]
baak1b18_at	7BS-ab-k71_contig_3100652:1-380	2E-92	#N/A
Contig15259_at	7BS-ab-k71_contig_3156232:805-2519	3E-37	gi|60100238|gb|AAX13288.1| senescence-associated protein [Triticum aestivum]
HVSMEn0021L12r2_at	2BS-ab-k71_contig_5166420:1548-4919	2E-08	#N/A
baak1b18_x_at	7BS-ab-k71_contig_3100652:1-380	2E-92	#N/A
HVSMEg0006C07r2_at	2AL-ab-k71_contig_6363708:6428-7130	2E-58	gi|82780760|gb|ABB90549.1| putative membrane protein [Triticum aestivum]
Contig18687_at	5BL-ab-k71_contig_10866110:23976-25433	3E-61	gi|475581852|gb|EMT18963.1| hypothetical protein F775_26051 [Aegilops tauschii]
Contig1012_s_at	5BL-ab-k71_contig_10834918:4913-6105	0	gi|475614191|gb|EMT29003.1| Chlorophyll a-b binding protein, chloroplastic [Aegilops tauschii]
Contig950_at	5AL-ab-k95_contig_1127310:1-799	1E-111	gi|131176|sp|P13194.2|PSAE_HORVU RecName: Full=Photosystem I reaction center subunit IV, chloroplastic; Short=PSI-E; AltName: Full=Photosystem I 10.8 kDa polypeptide; Flags: Precursor gi|19087|emb|CAA68782.1| unnamed protein product [Hordeum vulgare] gi|326503110|dbj|BAJ99180.1| predicted protein [Hordeum vulgare subsp. vulgare] gi|226163|prf||1413233A 10.8kD photosystem I protein
Contig2859_s_at	2AL-ab-k71_contig_6363708:6428-7130	3E-98	gi|82780760|gb|ABB90549.1| putative membrane protein [Triticum aestivum]
Contig926_x_at	6BS-ab-k71_contig_2981828:642-1842	0	gi|473952980|gb|EMS50796.1| Chlorophyll a-b binding protein, chloroplastic [Triticum urartu]
Contig26393_at	2BL-ab-k71_contig_7955957:1964-3724	1E-169	gi|326527993|dbj|BAJ89048.1| predicted protein [Hordeum vulgare subsp. vulgare]
Contig2411_at	2AS-ab-k71_contig_5190107:3940-11214	1E-134	gi|357122526|ref|XP_003562966.1| PREDICTED: uncharacterized protein LOC100831317 [Brachypodium distachyon]
Contig168_at	4AL_v2-ab-k71_contig_4AL_5754445:1016-3177	0	gi|473913339|gb|EMS49688.1| Chlorophyll a-b binding protein CP26, chloroplastic [Triticum urartu]
Contig960_s_at	6AS-ab-k71_contig_4354575:1792-2909	1E-116	gi|473952980|gb|EMS50796.1| Chlorophyll a-b binding protein, chloroplastic [Triticum urartu]
HO08B11S_at	4AL_v2-ab-k71_contig_4AL_7099598:762-2473	1E-44	gi|475559215|gb|EMT13284.1| 3-ketoacyl-CoA synthase 6 [Aegilops tauschii]

Table S5. List of 33 loci significantly upregulated between HD and 12 DAA in WT plants but not in *gpc-A1* plants.

Locus	IWGSC ID	Sequence description	Gene name
IWGSC_CSS_7AL_scaff_4381790:1-1789	Traes_7AL_6AE850114	Cadmium zinc-transporting ATPase 3	*TaHMA2-likeA*
IWGSC_CSS_7BL_scaff_6741649:1-1191	Traes_7BL_0CF58CF4E	Cadmium zinc-transporting ATPase 3	*TaHMA2-likeB*
IWGSC_CSS_3AS_scaff_3289235:1-527	Traes_3AS_E59FB52EC	Probable peptide nitrate transporter at3g43790-like	*TaZIFL2-like1A*
IWGSC_CSS_4BS_scaff_4884666:3611-13951	Traes_4BS_1DCF82CB7	Protein zinc induced facilitator-like 1-like	*TaZIFL3B*
IWGSC_CSS_7AL_scaff_4404279:2840-3532	Traes_7AL_F1D611563	Zinc transporter 10	*TaZIP10-like1A*
IWGSC_CSS_2BS_scaff_5173470:2532-5129	Traes_2BS_9D8F265EC	Zinc transporter 1	*TaZIP13-like2B*
IWGSC_CSS_2BL_scaff_8072119:162-3787	Traes_2BL_A1BCBD2BE	Zinc transporter 1	*TaZIP13-likeB*
IWGSC_CSS_2AL_scaff_1213723:1-2131	Traes_2AL_3983FD077	Zinc transporter zip1	*TaZIP3A*
IWGSC_CSS_1AS_scaff_3313103:14-652	Traes_1AS_EC8891094	Zinc transporter protein zip7	*TaZIP7A*
IWGSC_CSS_1AS_scaff_3258126:5939-9519	Traes_1AS_50323685E	Zinc transporter 7-like	*TaZIP7A*
IWGSC_CSS_1BS_scaff_3424485:2111-5600	Traes_1BS_D68F0BED6	Zinc transporter 7-like	*TaZIP7B*
IWGSC_CSS_7BS_scaff_3097754:654-17033	Traes_7BS_BB0FECDBE	ABC transporter C family member 10	
IWGSC_CSS_3AS_scaff_3280126:1-370	N/A	Aquaporin TIP4-3-like	
IWGSC_CSS_3B_scaff_7192099:7-5760	N/A	Coatomer subunit beta -1	
IWGSC_CSS_2BL_scaff_8006213:433-3492	Traes_2BL_ACB3018DD	Ferredoxin--NADP embryo chloroplastic	
IWGSC_CSS_2AL_scaff_6420325:2830-4395	Traes_2AL_D24C65691	Ferredoxin--NADP embryo chloroplastic	
IWGSC_CSS_4BS_scaff_4865990:1587-2676	Traes_4BS_FFF12BA9E	Ferritin 2a	
IWGSC_CSS_2BL_scaff_8011889:2013-8385	Traes_2BL_F2772688F	GDSL esterase lipase EXL3	
IWGSC_CSS_1BL_scaff_814680:1-755	Traes_1BL_8DAC11230	**Inwardly rectifying potassium channel AKT2**	
IWGSC_CSS_5AL_scaff_2808642:7307-9079	Traes_5AL_6C8BD96CB	Nucleolar protein 58-like isoform x1	
IWGSC_CSS_6AL_scaff_5832728:1-551	N/A	Peroxisomal multifunctional enzyme type 2	
IWGSC_CSS_6BL_scaff_3861072:390-904	N/A	Peroxisomal multifunctional enzyme type 2-like	
IWGSC_CSS_7AL_scaff_4506504:232-7123	Traes_7AL_7BD10B6C7	Pleiotropic drug resistance protein 12-like	
IWGSC_CSS_2BS_scaff_5172493:6780-8736	Traes_2BS_D5A97B888	Polyol transporter 5-like	
IWGSC_CSS_5AL_scaff_2750252:1939-3125	Traes_5AL_8F16AB90F	Probable auxin efflux carrier component 4-like	
IWGSC_CSS_2BL_scaff_8087489:9565-11148	Traes_2BL_0E87D8729	Probable peptide nitrate transporter at1g22540-like	
IWGSC_CSS_3B_scaff_10437122:613-1435	Traes_3B_F81203C21	Protein HASTY1-like	
IWGSC_CSS_1AL_scaff_2791900:7869-11173	Traes_1AL_039C1D96D	Protein RUPTURED POLLEN GRAIN 1	
IWGSC_CSS_1AL_scaff_3915680:8466-19572	Traes_1AL_C921492AA	Squamosa promoter-binding-like protein 9	
IWGSC_CSS_3B_scaff_10770864:1-1807	Traes_3B_F6D9A9AEE	S-type anion channel SLAH3	
IWGSC_CSS_4AL_scaff_495051:405-909	Traes_4AL_0FEA52671	**Sulfate transporter**	
IWGSC_CSS_2AL_scaff_6345426:2122-3685	N/A	**Sulfate transporter**	
IWGSC_CSS_1BL_scaff_3829677:1628-1984	Traes_1BL_CF65BE819	Zinc transporter 9-like	

Table S6. Details of field experiments.

Experiment	UCD-2012	TAU-2012	NY-2012	NY-2013
**Location**	Davis, CA, 38° 32 ′N, 121° 46′ W	Tel-Aviv University 32° 6’N 34° 48’E	Newe Ya'ar Regional Research Center 32° 42’N 35° 10’E	Newe Ya'ar Regional Research Center 32° 42’N 35° 10’E
**Planting date**	11/23/2011	12/12/2011	12/7/2011	12/2/2012
**Sowing**	50 seeds per row	8 pre-germinated seedlings per row	8 pre-germinated seedlings per row	8 pre-germinated seedlings per row
**Soil**	Yolo loam soil (fine silty, mixed, superactive, nonacid, thermic Mollic Xerofluvent)	Hamra soil (90% sand, 10% clay, soil N 8.2 mg kg^{-1})	Heavy soil (18.7% inorganic matter, 22.6% sand, 57.0% silt, 1.7% clay and organic matter)	Heavy soil (18.7% inorganic matter, 22.6% sand, 57.0% silt, 1.7% clay and organic matter)
**Precipitation during growing season (mm)**	232	522	566	682
**Irrigation**	1 acre foot (326,000 gallons) added 05/17/2012	-	-	-
**Fertilizer**	11g/row ammonium sulfate added 2/13/2012	100 kg ha^{-1} basal N fertilization	100 kg ha^{-1} basal N fertilization	100 kg ha^{-1} basal N fertilization
**Randomized complete blocks**	10	4	5	4

Table S7. qRT-PCR primers used in this study and their efficiency.

Target	F primer	R primer	Efficiency (%)
HMA2-like	GAGATCCATGGGCATCAAGT	CTTCCGGCAAAAGTTCAGAG	97.4%
NAAT2	ACCCAGCAACCTTCATTCAG	CAGACCGATAATCCCCTTGA	95.5%
ZIP10-like1	GCCTTCTTCTTTGCCATCAC	GGGCTGTATGGGTCGTAGAA	95.0%
ZIP10	GTGGATCTCATTGCTGCTGA	AGCCCAAATAGCCAGTGATG	95.8%
ZIP3	CTTTGAAGGCATAGGCTTGG	GATGGTTGCCCTTACCTTGA	95.3%
ZIP7	CAGGGCTCTAGTGGTCGAAG	AGAAATCGGCTGCGATTAGA	97.3%
